# Supplementary material for: HOXBLINC long non-coding RNA activation promotes leukemogenesis in NPM1-mutant acute myeloid leukemia
Source: Nat Commun. 2021 Mar 29;12:1956. doi: 10.1038/s41467-021-22095-2 (PMC8007823; doi:10.1038/s41467-021-22095-2)
Supplement: Supplementary file 1 — Supplementary Information [file 41467_2021_22095_MOESM1_ESM.pdf]

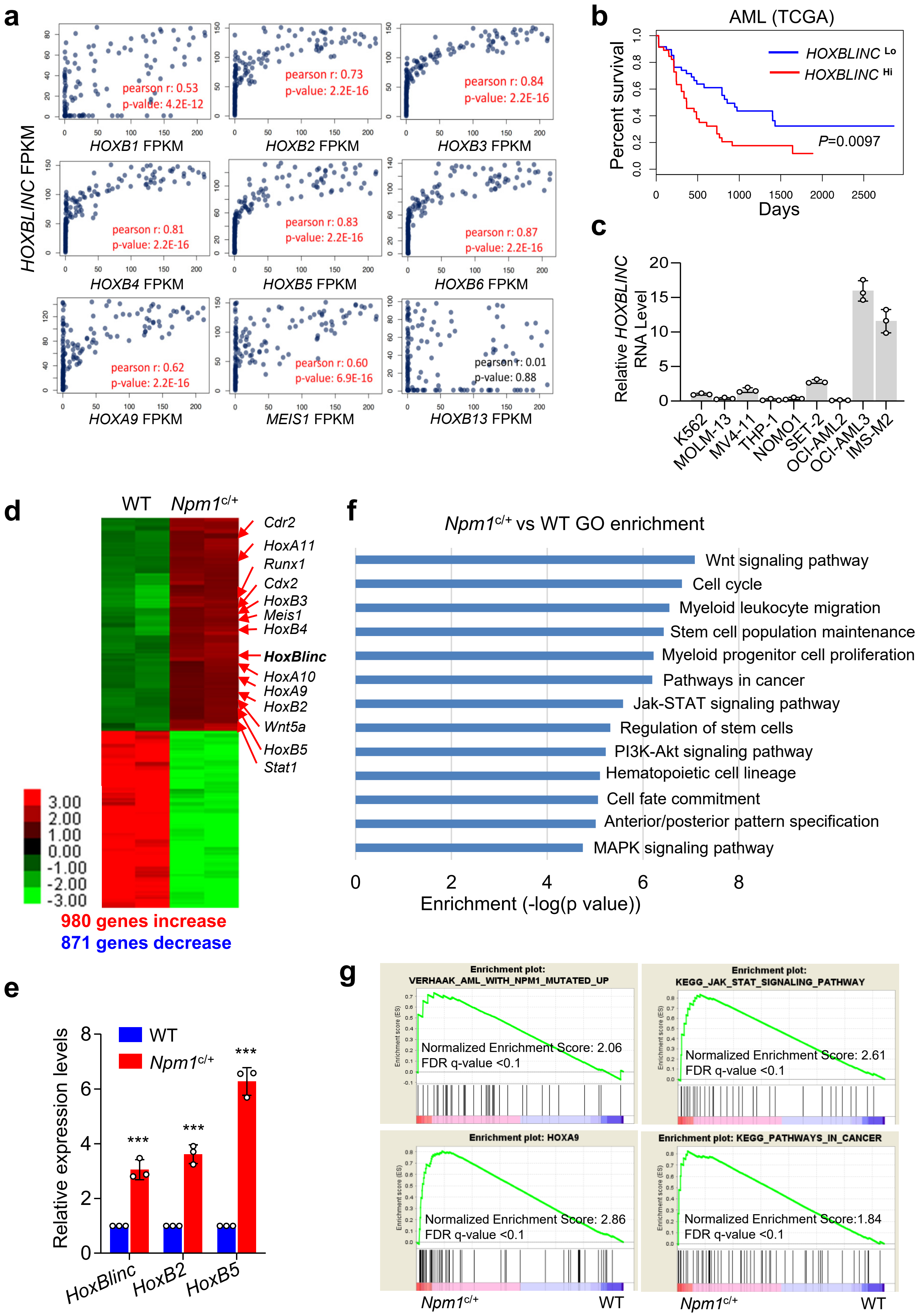

Zhu et al. Supplementary Figure 1

Supplementary Figure 1, related to Figure 1. a Correlation between the expression of *HOXBLINC* and anterior *HOXB* genes, *HOXA9*, *MEIS1* and *HOXB13* in the TCGA AML datasets (GSE62944). Pearson's *r* and corresponding *p* value were calculated by the cor.test of R. **b** Survival analysis in AML patients (*n*=179) of the AML TCGA dataset stratified by *HOXBLINC* expression. $HOXBLIN^{Hi}$, the top thirty percentile of AML patients based on *HOXBLINC* expression; $HOXBLINC^{Lo}$, the bottom thirty percentile (n=49/group). The Log-rank test was used to analyze differences between the survival curves. **c** RT-qPCR analyses of *HOXBLINC* expression levels in different AML cell lines (cell line information is listed in Table S8) with various gene mutations (Data is presented as mean ± SD, n=3 independent experiments). **d** Heatmap of RNA-seq analysis shows the up- and down-regulated genes in *Npm1*c^{+} knock in (KI, $Npm1^{c/+}$) vs. WT LSK cells based on 2 independent experiments. Red arrows: up-regulated genes implicated in HSPC regulation and/or leukemogenesis. **e** RT-qPCR analysis of *HoxBlinc*, *HoxB2* and *HoxB5* expression levels in WT and $Npm1^{c/+}$ LSK cells (Bars represent mean ±SD; ***P<0.001 by two-tailed unpaired Student's *t*-test; n=3 by three independent experiments). **f** The *Npm1*c^{+} KI affected genes in LSK cells were analyzed and annotated by the Gene Ontology (GO) analysis. **g** Enrichment of *Npm1*c^{+} KI dysregulated genes in AML with *NPM1* mutated, HOXA9, JAK-STAT, and Cancer pathways by Gene Set Enrichment Analysis (GSEA).

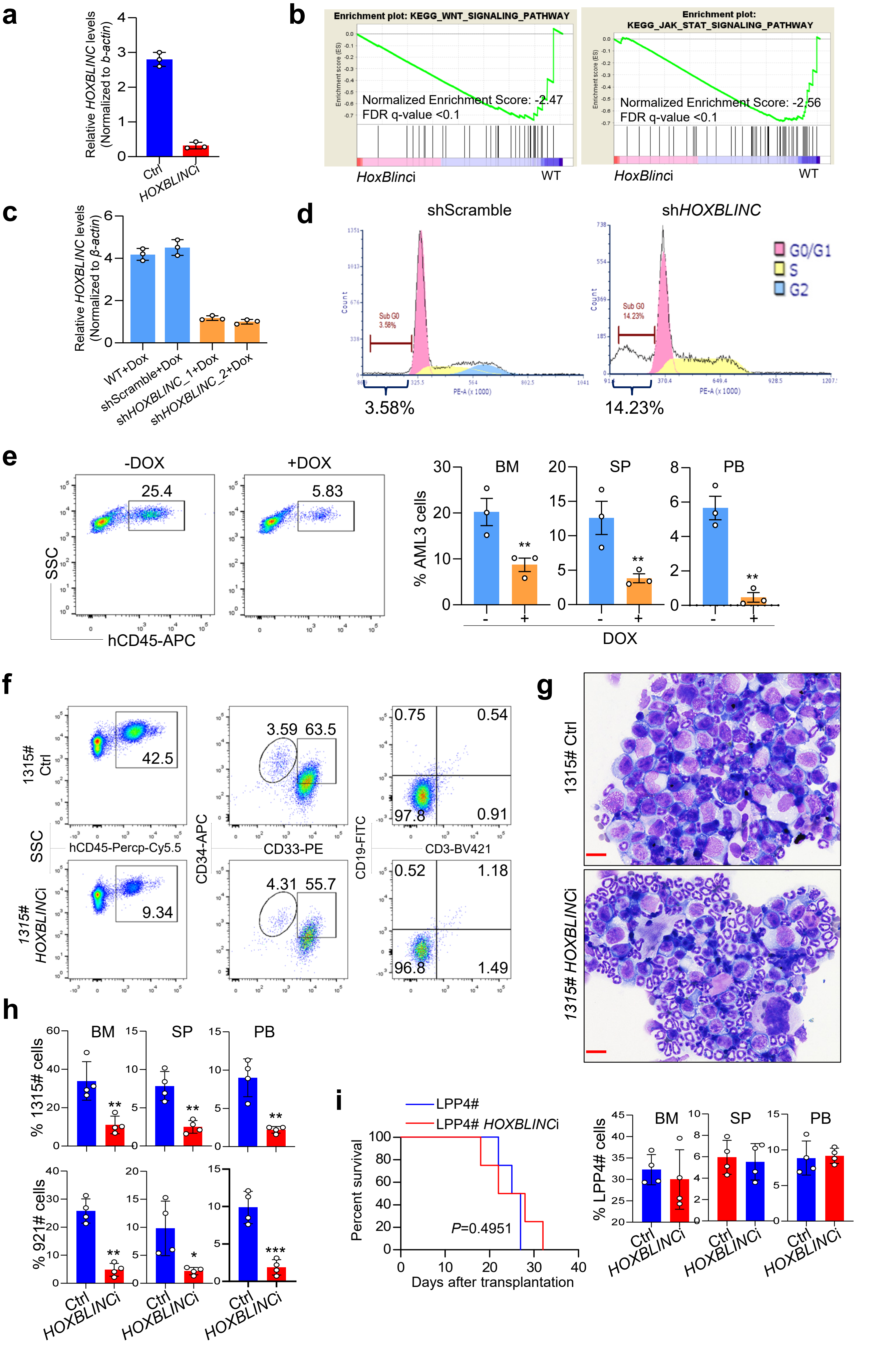

Zhu et al. supplementary Figure 2

Supplementary Figure 2, related to Figure 1. *HOXBLINC* is critical for maintaining the leukemic state of *NPM1c*$^{+}$ AML. a RT-qPCR analysis of *HOXBLINC* levels in WT and *HOXBLINC*-dCas9-KRAB OCI-AML3 cells to determine the *HOXBLINC* inhibition efficiency. Bars represents mean ± SD from three independent experiments. **b** Enrichment of *HOXBLINC*-KRAB dysregulated genes in WNT (*left*) and JAK-STAT (*right*) signaling pathways by GSEA. **c** RT-qPCR analysis of *HOXBLINC* levels in WT, sh*Scramble*, sh*HOXBLINC*_1#, and sh*HOXBLINC*_2# OCI-AML3 cells after Dox treatment to determine the *HOXBLINC* knockdown (KD) efficiency based on three independent experiments (Bars represent mean ± SD). **d** FACS analysis of cell cycle using propidium iodide shows that *HOXBLINC* KD increases the sub-G0 cell population in OCI-AML3 cells as compared to *shScramble* cells. **e** FACS analysis of human CD45^{+} cell chimerism in bone marrow (BM), spleen (SP), and peripheral blood (PB) cells of recipient NSG mice at 30 days after receiving *shHOXBLINC* OCI-AML3 cells. Animals were treated with or without doxycycline (Dox). n=3 mice/group were analyzed by FACS. Data is presented as mean ± SEM; ***P*<0.01 by two-tailed unpaired Student's t-test. **f** Representative scatter plots of the FACS analyses for the human AML cell engraftment in the BM of recipient mice transplanted with 1315# patient BMs. **g** Representative images of May-Giemsa stained cytospins of BM cells prepared from 4 control and 4 *HOXBLINCi* 1315# recipient mice. Scale bar, 20µm. **h** hCD45^{+} cell chimera in BM, SP, and PB of mice transplanted with control or *HOXBLINCi* BM cells from AML patients with *NPM1*c^{+} mutations. 1315# (*NPM1*c^{+};*FLT3*wt, *top*), 921# (*NPM1*c^{+};*FLT3*mu, *bottom*) (n=3 mice/group were analyzed by FACS; bars represents mean ± SD; **P*<0.05, ***P*<0.01, ****P*<0.001 by two-tailed unpaired Student's t-test.). **i** Kaplan-Meier survival curves (*left*) and hCD45+ cell chimera in BM, SP, and PB (*right*) of mice transplanted with control or *HOXBLINCi* BM cells from an AML patient (LPP4#, *NPM1*wt;*MLL*r+) without *NPM1* mutation (n=4 mice/group were analyzed by FACS; bars represents mean ± SD; statistical significance is calculated by two-tailed unpaired Student's t-test.)

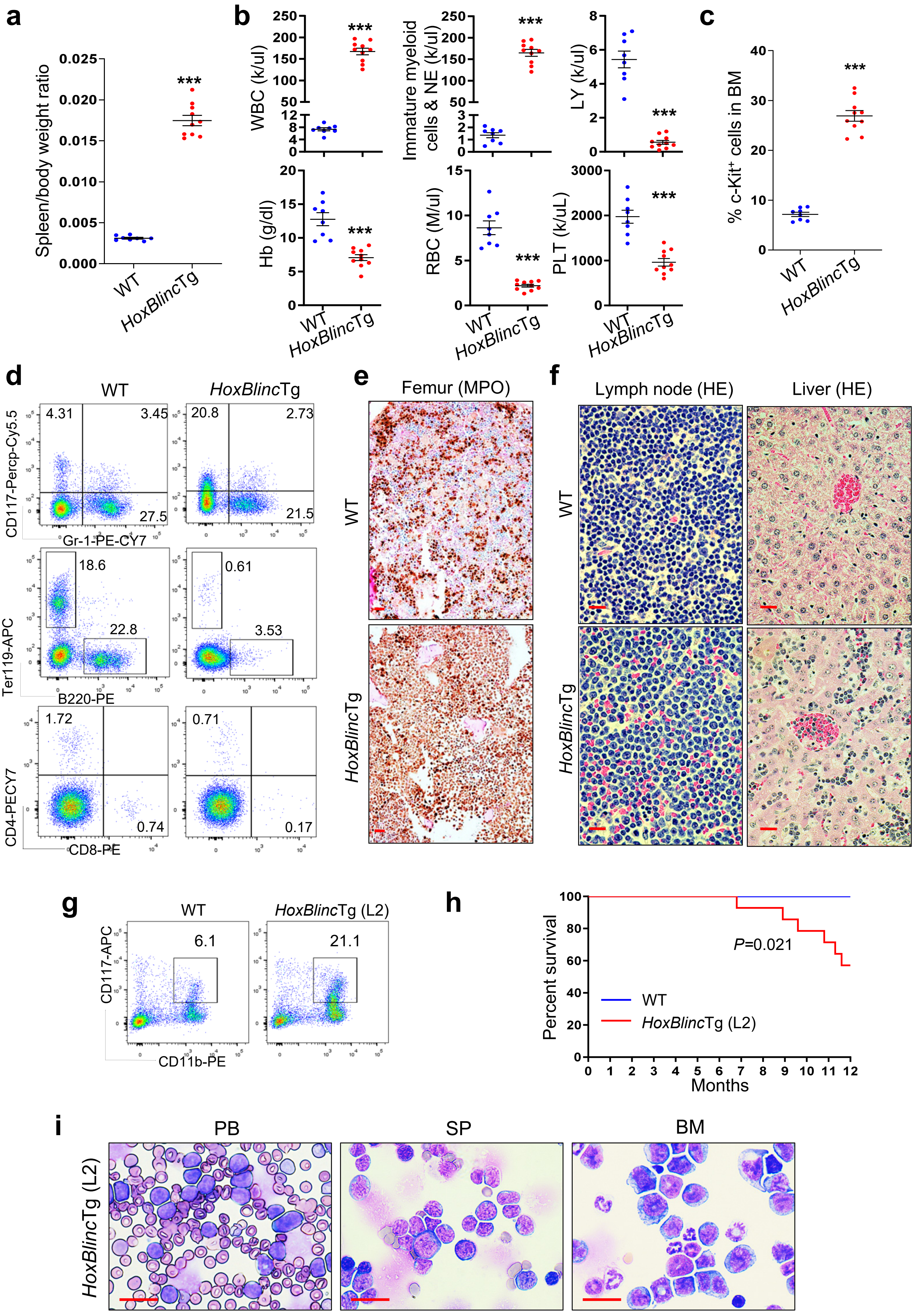

a
Spleen/body weight ratio
0.025
0.020
0.015
0.010
0.005
0.000
***
WT
HoxBlincTg
b
WBC (k/ul)
Immature myeloid cells & NE (k/ul)
LY (k/ul)
Hb (g/dl)
RBC (M/ul)
PLT (k/uL)
***
c
% c-Kit+ cells in BM
d
WT
HoxBlincTg
CD117-Percp-Cy5.5
Gr-1-PE-CY7
4.31
3.45
27.5
20.8
2.73
21.5
Ter119-APC
B220-PE
18.6
22.8
0.61
3.53
CD4-PECY7
CD8-PE
1.72
0.74
0.71
0.17
e
Femur (MPO)
f
Lymph node (HE)
Liver (HE)
g
WT
HoxBlincTg (L2)
CD117-APC
CD11b-PE
6.1
21.1
h
Percent survival
P=0.021
WT
HoxBlincTg (L2)
Months
i
PB
SP
BM
HoxBlincTg (L2)

Supplementary Figure 3, **related to Figure 2. Development of AML in *HoxBlinc*Tg mice.** **a** Spleen/body weight ratio for age-matched WT (n=8) and diseased *HoxBlinc*Tg (n=10) mice. **b** Parameters of PB counts were summarized from diseased *HoxBlinc*Tg (n=10) and age-matched WT (n=8) mice. WBC: white blood cells, NE: neutrophils, LY: lymphocytes, Hb: hemoglobin, RBC: red blood cells, PLT, platelets. **c** Quantitation of c-Kit$^+$ cells within total BM cells of age-matched WT (n=8) and diseased *HoxBlinc*Tg (n=10) mice. Data in (**a**-**c**) are presented as mean ± SEM, ***P<0.001 by two-tailed unpaired Student's *t*-test. **d** Representative FACS analysis of c-Kit/Gr-1 (*top*), Ter119/B220 (*middle*) and CD4/CD8 (*bottom*) cell populations in the BM of diseased *HoxBlinc*Tg and age-matched WT mice. **e** Representative images of MPO stained femur sections from 3 WT and 4 moribund *HoxBlinc*Tg mice. **f** Representative images of H&E stained lymph nodes and liver sections of 6 diseased *HoxBlinc*Tg and 6 WT mice. **g** Representative FACS analysis of c-Kit$^+$/CD11b$^+$ cell populations in the BM of diseased Line#2 *HoxBlinc*Tg (L2) and age-matched WT mice. **h** Kaplan-Meier survival curve of WT (n=10) and Line#2 *HoxBlinc*Tg (n=14) mice up to 1 year of age. *P* value was calculated by Log-rank test. **i** Representative images of May-Giemsa stained PB smears, and cytospins of BM and spleen (SP) cells prepared from 4 moribund Line#2 *HoxBlinc*Tg mice (Scale=20µm).

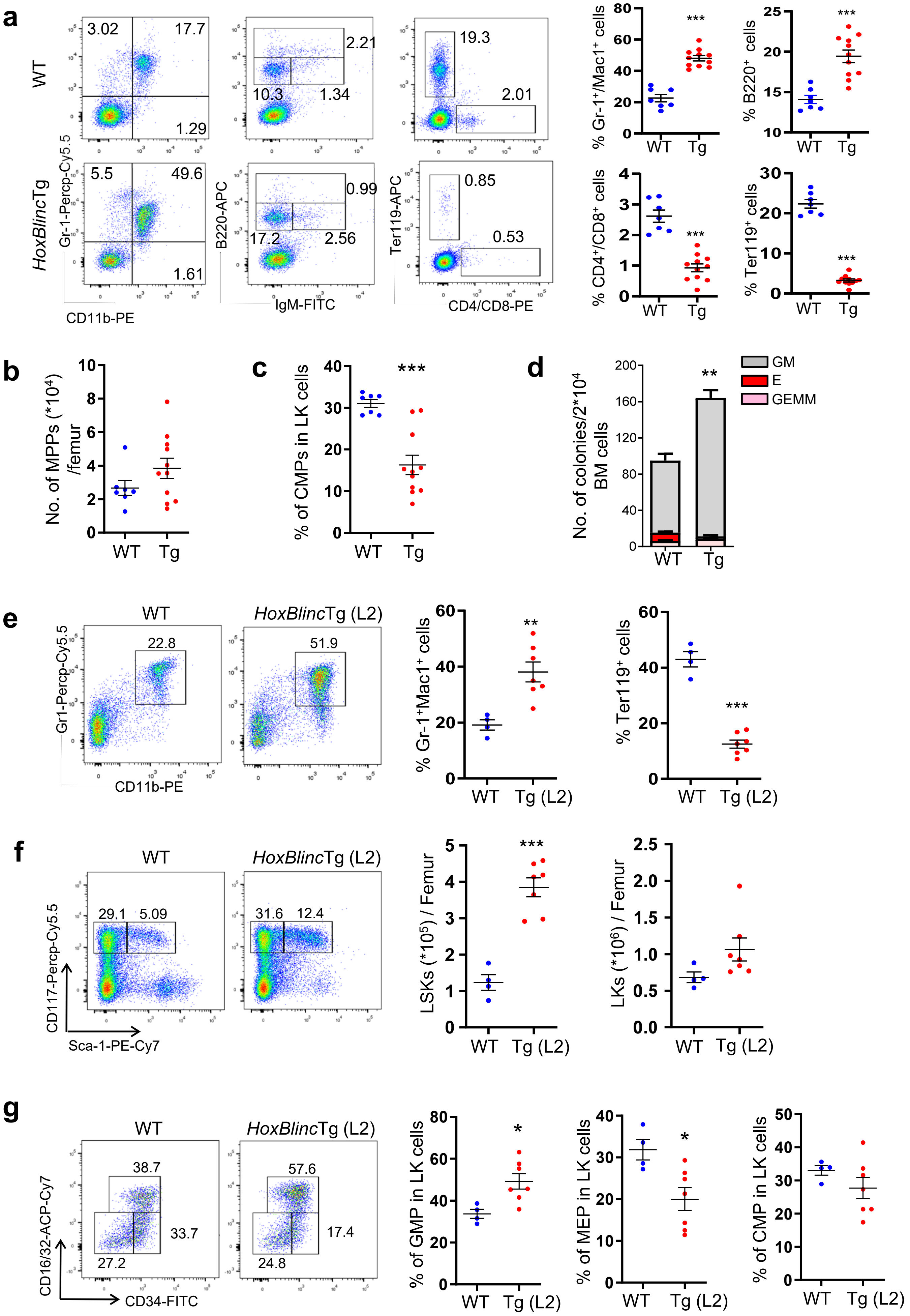

Zhu et al. Supplementary Figure 4

Supplementary Figure 4, **related to Figure 3. Transgenic expression of *HoxBlinc* enhances HSC self-renewal and expands myelopoiesis. a** Quantitation of the percentage of the Gr-1^+/Mac1^+, B220^+, CD4^+/CD8^+, and Ter119^+ cells in the BM of young WT (n=7) and *HoxBlinc*Tg (n=11) mice (8-10 weeks old) as determined by flow cytometry analysis. FACS plots from representative WT and *HoxBlinc*Tg mice are shown at left panel. **b** Quantitation of the total number of MPP cells per femur from these young WT (n=7) and *HoxBlinc*Tg (n=11) mice. **c** Quantitation of the percent CMP cells in the BM LK cells from these young WT (n=7) and *HoxBlinc*Tg (n=11) mice. **d** Frequencies of CFU-Cs in the BM cells from WT and *HoxBlinc*Tg mice (n=3/genotype by three independent experiments). GM: granulocytes/macrophages; E: burst forming unit-erythrocyte; GEMM: mixed colonies of GM and E and/or megakaryocytic cells. (**e**-**g**) FACS analysis of myeloid (Gr-1/CD11b), **e**: LSK/LK (Lin$^-$ cell gated); **f**: and GMP/CMP/MEP (LK cell gated); **g**: cell populations in BM of WT (n=4) and Line#2 *HoxBlinc*Tg (L2, n=7) mice (8-12 weeks old). Data in (**a**-**g**) are presented as mean ± SEM, *P<0.05, **P<0.01, ***P<0.001 by two-tailed unpaired Student's *t*-test.

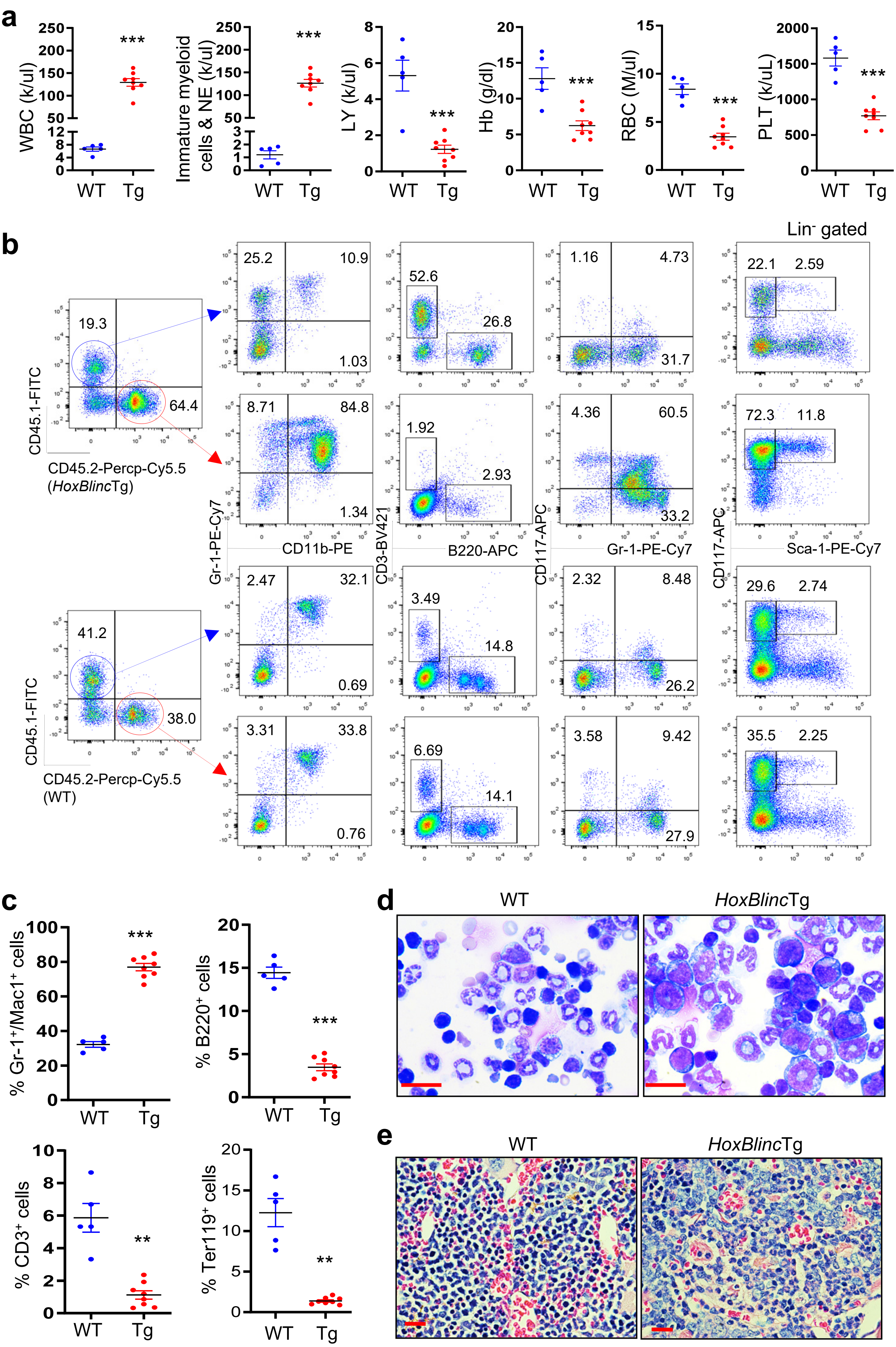

Zhu et al. Supplementary Figure 5

Supplementary Figure 5, related to Figure 3. The aberrant HSPC function and AML-like disease induced by *HoxBlinc* overexpression in mice are transferable. a Parameters of PB counts were summarized from recipient mice transplanted with WT (n=5) or *HoxBlinc*Tg (n=8) BM cells (Bars represents mean ± SEM; ***P<0.001 calculated by two-tailed unpaired Student's t-test.). **b** Flow cytometric analyses showing CD45.2 vs. CD45.1 chimerism as well as their respective lineage distribution (Gr-1/CD11b, CD3/B220, Gr-1/c-Kit) and percent LSK/LK cell populations (within Lin^- cells) in the BM of representative mice receiving WT or *HoxBlinc*Tg BM cells. **c** Quantitation of the percent lineage cell populations in the BM $CD45.2^+$ cell populations from recipient mice transplanted with WT (n=5) or *HoxBlinc*Tg (n=8) BM cells (Bars represents mean ± SEM; **P<0.01, ***P<0.001 calculated by two-tailed unpaired Student's t-test.). **d** Representative images of May–Grunwald–Giemsa stained cytospin preparations of BM cells from 3 mice receiving WT or *HoxBlinc*Tg BM cells. **e** Representative images of H&E stained femur sections of 3 recipients receiving WT or *HoxBlinc*Tg BM cells. Scale bar, 20μm. Data in (**a**, **c**) are presented as mean ± SEM, **P<0.01, ***P<0.001 by two-tailed unpaired Student's *t*-test.

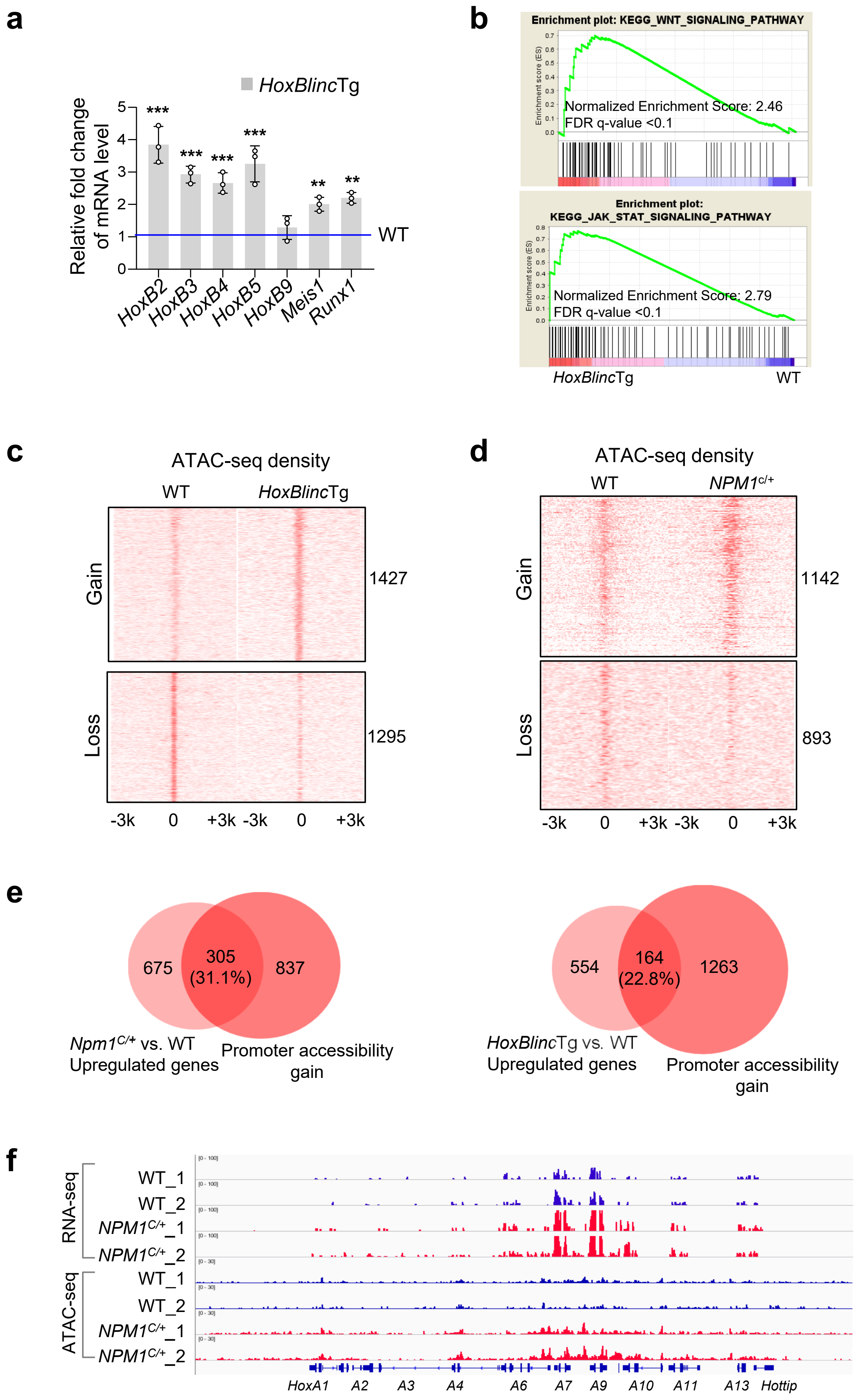

Zhu et al. supplementary Figure 6

g

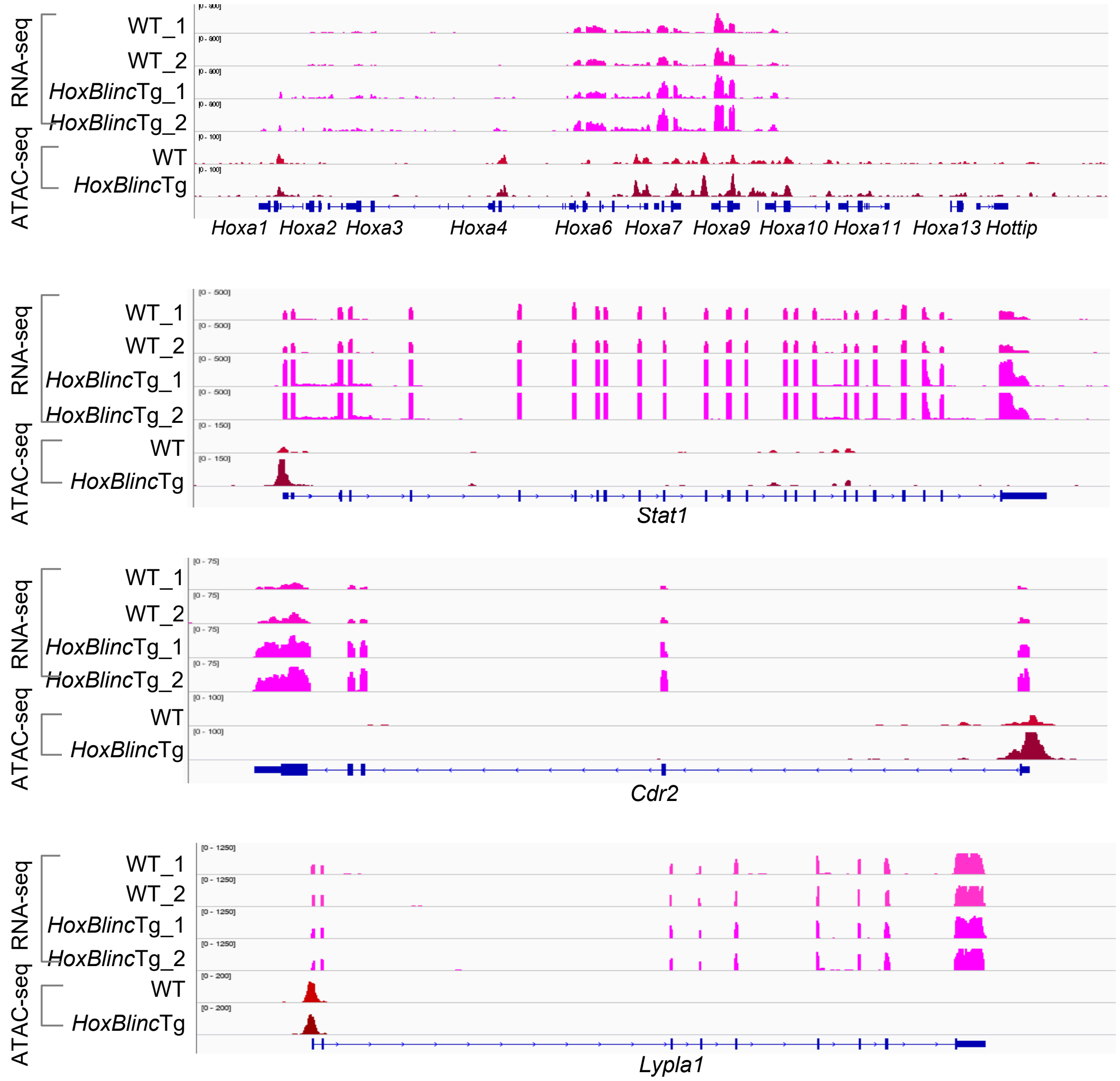
RNA-seq
ATAC-seq
WT_1
WT_2
HoxBlincTg_1
HoxBlincTg_2
WT
HoxBlincTg
Hoxa1 Hoxa2 Hoxa3 Hoxa4 Hoxa6 Hoxa7 Hoxa9 Hoxa10 Hoxa11 Hoxa13 Hottip
Stat1
Cdr2
Lypla1

Supplementary Figure 6, related to Figure 4. *Npm1*c^{+} knock in or transgenic expression of *HoxBlinc* remodels chromatin structure and alters hematopoietic transcription programs. a RT-qPCR analysis shows the relative mRNA levels of *HoxB2-5*,9, *Meis1* and *Runx1* genes in *HoxBlinc*Tg LSK cells to those in WT (Bars represent mean±SD; ***P*<0.01, ****P*<0.001 by two-tailed unpaired Student's *t*-test; n=3 by three independent experiments). **b** Enrichment of upregulated genes involved in WNT (*Top*) and JAK-STAT (*Bottom*) signaling pathways upon overexpression of *HoxBlinc* in LSK cells by GSEA. **c** ATAC-seq promoter density map of WT and *HoxBlinc*Tg LSK cells. **d** ATAC-seq promoter density map of WT and *Npm1*$^{c/+}$ LSK cells. **e** Overlap of upregulated genes and genes with promoter accessibility gain based on RNA-seq and ATAC-seq datasets of *NPM1*$^{c/+}$ vs. WT (*right*) and *HoxBlinc*Tg vs. WT (*left*) LSK cells. **f** RNA-seq (chromatin accessibility, *top 4 tracks*) and ATAC-seq (gene expression, *bottom 4 tracks*) analysis of WT and *Npm1*$^{c/+}$ LSK cells in the *HoxA* gene locus. **g** RNA-seq analysis (*top 4 tracks*) and ATAC-seq analysis (*bottom 2 tracks*) of WT and *HoxBlinc*Tg LSK cells in the *HoxA*, *Stat1*, *Cdr2* and *Lypla1* gene loci.

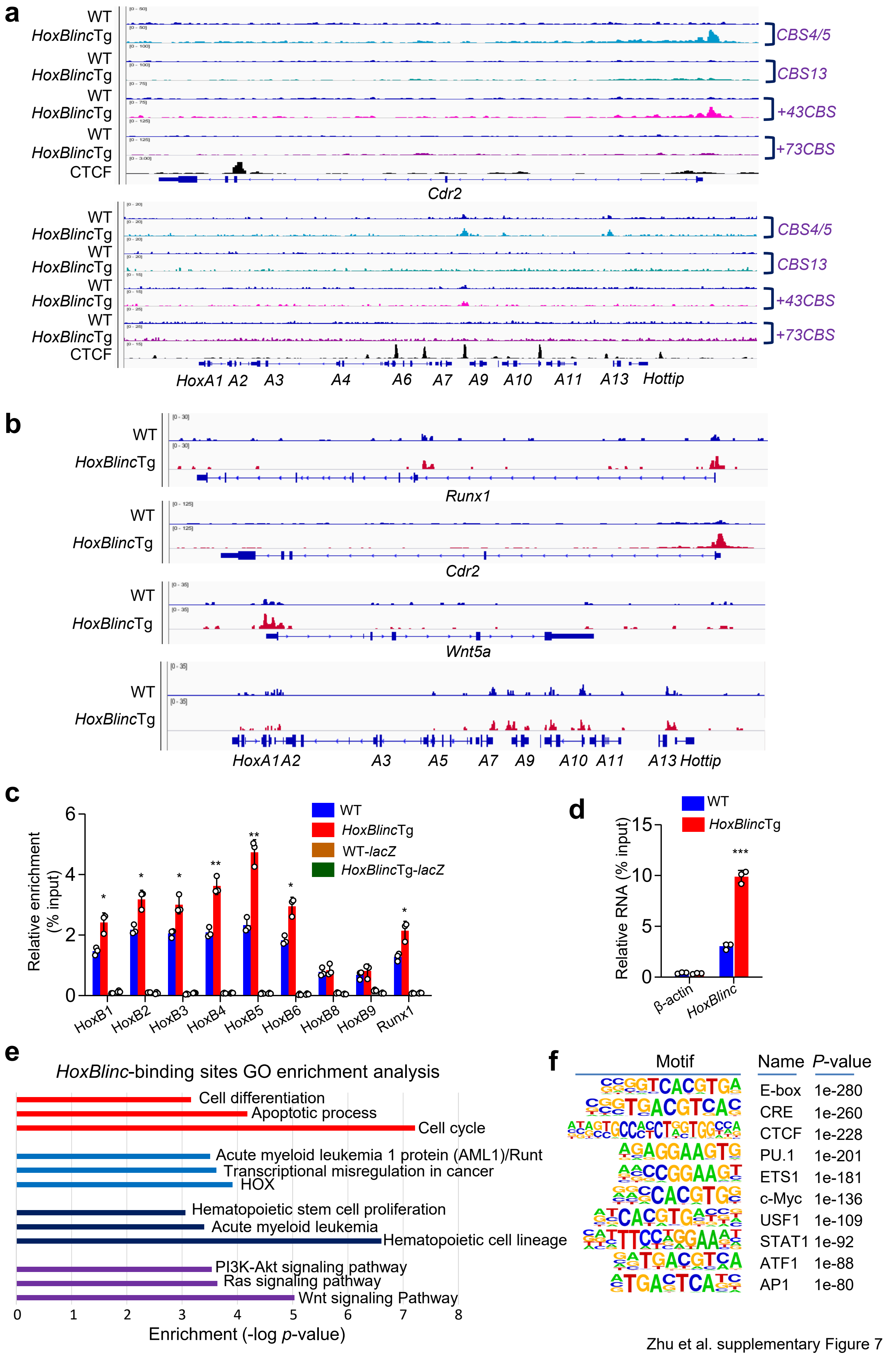

Zhu et al. supplementary Figure 7

Supplementary Figure 7, related to Figure 5. Transgenic expression of *HoxBlinc* alters long-range chromatin interactions and enhancer/promoter accessibility in Lin$^-$c-Kit$^+$ cells. a Long-range chromatin interactions (viewed from 4 different baits of *HoxB* loci) with *Cdr2* and *HoxA* loci as determined by 4C-seq analysis in WT and *HoxBlinc*Tg Lin$^-$c-Kit$^+$ cells. *HoxBlinc* overexpression increased the interactions of CBS4/5 and +43CBS with the *Cdr2* (*top*) and posterior *HoxA* (*bottom*) genes. ChIP-seq analysis of CTCF binding sites was obtained from the NCBI GEO public database (GSM918748). **b** ChIRP-seq analysis of *HoxBlinc* bindings to the *Cdr2*, *Wnt5a* and *HoxA* gene loci in WT and *HoxBlinc*Tg Lin$^-$c-Kit$^+$ cells. **c** ChIRP-qPCR analysis of the bindings of *HoxBlinc* to the promoter regions of selected genes in WT and *HoxBlinc*Tg Lin$^-$c-Kit$^+$ cells. The bindings of *HoxBlinc* to the *HoxB1-6* and *Runx1* promoter regions are enhanced by *HoxBlinc* overexpression in Lin$^-$c-Kit$^+$ cells. LacZ probe were used as negative control (Bars represent mean±SD; **P*<0.05, ***P*<0.01 by two-tailed unpaired Student's *t*-test based on the data of two independent experiments). **d** RT-qPCR analysis of RNA retrieved by the complementary *HoxBlinc* tiling probes in *HoxBlinc*Tg and WT Lin$^-$c-Kit$^+$ cells. (Bars represent mean ± SD; ****P*<0.001 by two-tailed unpaired Student's *t*-test from two independent experiments). **e** The genes which *HoxBlinc* binds to their promoter regions in Lin$^-$c-Kit$^+$ cells were analyzed and annotated by the Gene Ontology analysis. **f** List of top 10 significant transcription factor binding motifs enriched in the *HoxBlinc* binding sites in *HoxBlinc*Tg Lin$^-$c-Kit$^+$ cells.

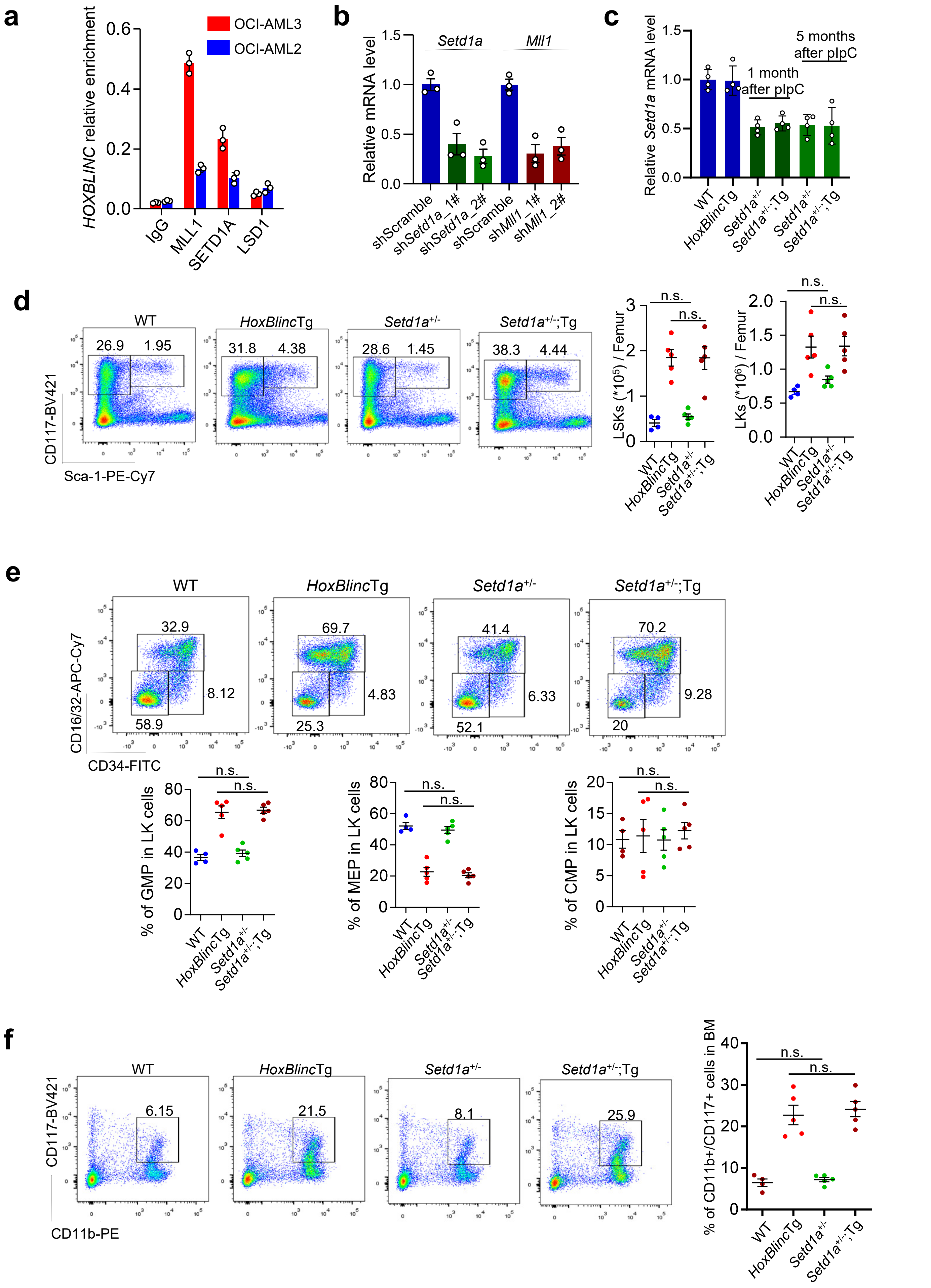
a
HOXBLINC relative enrichment
OCI-AML3
OCI-AML2
IgG
MLL1
SETD1A
LSD1
b
Relative mRNA level
Setd1a
Mll1
shScramble
shSetd1a_1#
shSetd1a_2#
shScramble
shMll1_1#
shMll1_2#
c
Relative Setd1a mRNA level
5 months after pIpC
1 month after pIpC
WT
HoxBlincTg
Setd1a+/-
Setd1a+/-;Tg
d
WT
HoxBlincTg
Setd1a+/-
Setd1a+/-;Tg
CD117-BV421
Sca-1-PE-Cy7
LSKs (*10^5) / Femur
LKs (*10^6) / Femur
n.s.
e
CD16/32-APC-Cy7
CD34-FITC
% of GMP in LK cells
% of MEP in LK cells
% of CMP in LK cells
f
CD117-BV421
CD11b-PE
% of CD11b+/CD117+ cells in BM

Supplementary Figure 8, **related to Figure 6.** ***Setd1a*** **is not a critical player in** ***HoxBlinc*** **overexpression mediated hematopoietic phenotypes. a** RIP-qPCR analysis of *HoxBlinc* RNA retrieved by antibodies against MLL1, SETD1A and LSD1 from OCI-AML3 and OCI-AML2 cells (bars represent mean ± SD; n = 3 by three independent experiments). **b** RT-qPCR analysis of *Setd1a* and *Mll1* mRNA levels in sorted GFP^{+}LSK cells transduced with Sh*Scramble*, sh*Setd1a* or sh*Mll1* lentivirus. Bars represent mean ± SD, n=3 by three independent experiments. **c** RT-qPCR analysis of *Setd1a* mRNA levels in the BM cells from *Setd1a*$^{+/-}$ or *Setd1a*$^{+/-}$;*HoxBlinc*Tg mice to determine the efficiency of pIpC for inducing *Setd1a* gene inactivation (Bars represent mean ± SD, n=3 mice/group). **d** FACS analysis pf LSK and LK cell populations in the BM Lin^{-} cells of WT, *HoxBlinc*Tg, *Setd1a*$^{+/-}$, and *Setd1a*$^{+/-}$;*HoxBlinc*Tg mice. Bars represent mean ± SEM. **e** FACS analysis of GMP, MEP and CMP populations within BM LK cells of WT, *HoxBlinc*Tg, *Setd1a*$^{+/-}$, and *Setd1a*$^{+/-}$;*HoxBlinc*Tg mice. Bars represent mean ± SEM. **f** FACS analysis of $CD117^{+}$/$CD11b^{+}$ cells in BM cells of WT, *HoxBlinc*Tg, *Setd1a*$^{+/-}$, and *Setd1a*$^{+/-}$;*HoxBlinc*Tg mice (Data in **d**-**f** are represented as mean ± SEM; n.s. means no significance by two-tailed unpaired Student's *t*-test; n=4 mice in WT, n=5 mice in *HoxBlinc*Tg, *Setd1a*$^{+/-}$, *Setd1a*$^{+/-}$;Tg).

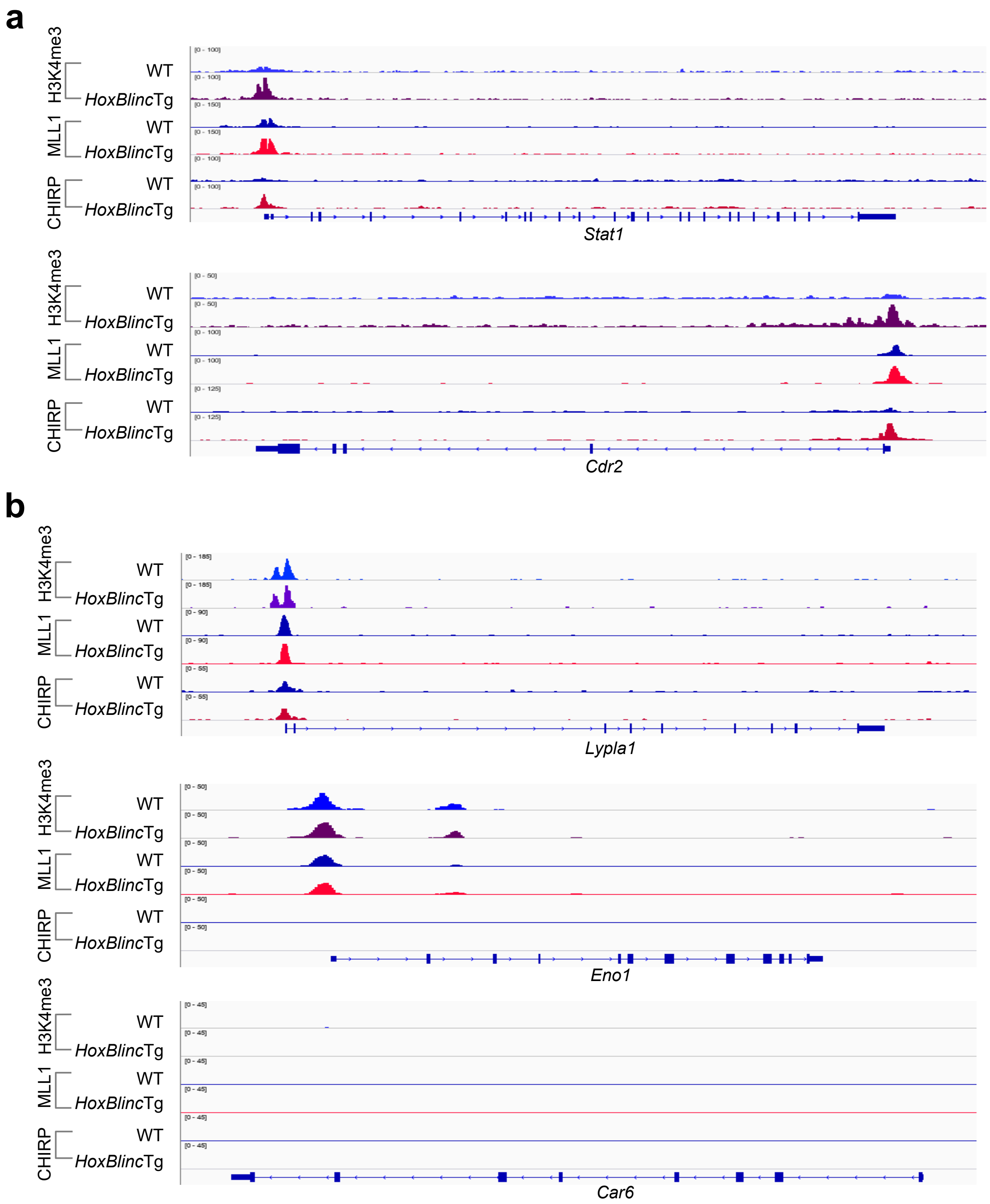

Zhu et al. supplementary Figure 9

Supplementary Figure 9, **related to Figure 6.** ***HoxBlinc*** **overexpression increases MLL1 recruitment and H3K4me3 occupancy to its target genes.** (**a**-**b**) ChIP-seq analysis of H3K4me3 (*top 2 tracks*), MLL1 (*middle 2 tracks*) and ChIRP-seq analysis of *HoxBlinc* (*bottom 2 tracks*) at the *Stat1, Cdr2, Lypla1, Eno1 and Car6* gene loci of WT and *HoxBlinc*Tg $Lin^{-}c\text{-}Kit^{+}$ cells.

Supplementary Table 1. Human AML patient information

**Patient samples used for *HOXBLINC* gene expression analysis**						
Sample#	AML Subtype	Mutation	Previous Treatment	Karyotype at Relapse	Response	Admission#
3	AML-M5b	DNMT3A R882P (47.40%); MYH11 R501H (53.25%)	Decitabine	47, XX, , +12[2]/47, idem, ?t (11; 19) (q23; p13.1)[17]/46, XX[1]	n/a	n/a
9	AML-M2a (MLL+)	IDH1 Exon6 (51.66%); FAT1 Exon27 (48.48%); TNFAIP3 Exon3 (100%); TP53 Exon9 (4.44%)	DA	46, XX[5]	n/a	n/a
11	AML-M2b (AML1/ETO+)	WT1 (34.85%)	DA	45, X, -Y, t (8; 21) (q22; q22)[20], AML1-ETO (+)	PR	n/a
12	AML-in (16) (p13q23)/CBFB-MYH11)	NRAS G13D; KRAS G35T; NOTCH2 I1689F; FAT1 V3719F; TNFAIP3 N102S; CRLF2 P224L; ASXL1 G652S	DA	47, XY, inv (16) (p13q22), +22[17]/46, XY[3]	CR	n/a
22	AML-M4EO (CBF-MYH11+)	KIT p.D816V, KRAS p.G13D, WT1 p.S381X, KRAS p.G12S	DA	46, XX[12]	CR	n/a
23	AML-M1 (MLL/AF6+, NR)	MLL-AF6 (+)	CAG, FLAG, DAC+MAC	47-51, XX, +?4, + 8, del (11) (q23), +?14, -?18, +23mar, inc[cp18]/46, XX[2]	NR	n/a
24	AML-M4Eo	KIT A2447T, FLT3 D835H, FLT3 N676K, JAK2 E890K, PTPN11 D61G	DA	46, XY, ? inv (16) (p13q22)[5]/46, XY[15]	CR	n/a
28	AML-M5b (NUP98-HOXA9+)	CBL (c.T1111C, c.A1112C), MUM1 (c.G1940A)	DA	46, XY, t (7; 11) (p15; p15)[20]	PR	n/a
32	AML-M4eo	NRAS G12D; TET2 F868L; FLT3 N676K; FAT1 E2496K; NF1 R416X; ASXL1G652S	DA	46, XX[20]	CR	n/a
33	AML-M4b (MLL-ELL)	NRAS G12V (2.45%); FLT3 V579A (2.46%); CSF3R P733T (96.64%); PRDM1P467L (47%); SETBP1R1466D (47.4%)	DA	46, XX, t (11; 19) (q23; p13)[20]/46, XX[1]	PR	n/a
37	AML-M5b	TET2 Exon3, ASXL1 Exon12	DA	CBL (c.T1111C, c.A1112C), MUM1 (c.G1940A)	CR	n/a
39	AML-M2b	SETBP1R627C, CSF3R P733T, CEBPA P23fs	DA	46, XX, t (8; 21) (q22; q22), del (9) (q22)[20]	PR	n/a
41	AML-M5a (MLL+)	IDH1; NOTCH1; KMT2D; CRLF2	IA+CAG	47, XY, +mar[5]/46, XY[4]	PR	n/a
44	AML-M5b	K-RAS; EP300; CSF3R; CREBBP;	DA	46, xx[20]	CR	n/a
50	AML-M5a	Negtive	DA	46, XX[10]	CR	n/a
51	AML-M5 (MLL-AF6)	FGFR3 G1285A 50.57%, KIT T84M 45.52%, ASXL1G1954A 53.32%	DA	46, XY, t (6; 11) (q27; q23)[20]	CR	n/a
53	AML-M4eo	KIT p.D816V 15.05%	DA	46, XX, inv (16) (p13q22)[12]/46, XX[8]	PR	n/a
60	AML-M5b	SH2D1A (51.2%); CSF3R (49.19%); TNFAIP3 (4.15%); PAX5 (5.95%); DIS3 (5.13%)	DA	46xx, t (9; 11) (p22; q23)[18]/46, xx	PR	n/a
11	AML-M2a	FLT3-ITD (10%), TET (Exon10, 47.9%), TET2 (Exon9, 36.5%)	DA	46, XX[20]	PR	n/a
12	AML-M5b (intermediate risk)	PTPN1： p.G60V： 22.9%; PTPN11p.A72V： 19.8%, other mutation:CALR： p.S214L： 45.4%	DA	46, XX[20]	CR	n/a
2	AML-M5b	NR'	DA	46, XY, t (7; 11) (p15; p15)	n/a	n/a
6	AML-M5 (MLL-ELL +)	BRAF-p.v600E; NRAS-p.G13D; NRAS-p.G12V; KRAS-p.G12V	DA	46, XY[20]	n/a	n/a
13	AML-M4	NOTCH2; DNM2	DA	42, X, -X, -8, -9, -11, -14, -16, +2mar, inc[1]/42, XX, -4, -8, -8, -9, -21, +mar, inc[1]/46, XX, -9, +mar, inc[1]/46, XX[17]	NR	n/a
14	AML-M5b	NRAS Q61H	IA	47, XX, +6, t (11; 19) (q23p13)[1]/46, XX, t (11; 19) (q23p13)[8]/46, XX[11]	n/a	n/a
16	AML- (CBFβ/MYH11+)	Negtive	MAE	46, XY[20]	n/a	n/a
17	AML-M4eo	NRAS; CSF3R; CYLD	DA	46, XY, 13ps+, inv (16) (p13q22)[20]	n/a	n/a
21	AML-M4eo (intermediate risky)	C-Kit	IA	46, XY[20]	CR	n/a
25	AML-M4/5?	NRAS Exon2 G13R, SETBP1 Exon6 P1563L	DA	46, XY, inv (16) (p13q22)[15]/46, XY[5]	CR	n/a
31	AML-M5b	NRAS p.G12D (38.48%), FAT1 p.Q587k (7.5%), TET2 p.Q324H (3.92%)	DA	46, XX, t (11; 19) (q23; p13)[16]/46, XX[4]	CR	n/a
2	AML-M5b	FLT3 ITD; NPM1 p.W88Cfs*12; HNRNPK; DIS3 p.E289; DNMT3A p.A368。	hydroxycarbamide, DA	46, XY[20]	PR	n/a
3	AML-M5b	FLT3-ITD; NPM1-c.860-863dupTCTG; TET2-R1216X; RELN-A1574T; FANCG-P590A	DA	46, XX	CR	n/a
4	AML-M5a	FLT3 ITD 60%; NPM1 p.W288Cfs*12 50%; DNMT3A p.R882s 50.6%; SETD2 p.S336X 48.5; ABL1 p.P899L 52.5%。NPM1: 51.68%。FLT3-ITD:0.75	DA	Normal	PR	n/a
5	AML-M5	FLT3 ITD 90%, DNMT3A Exon23 c.2645G>A p.R882H rs147001633 53.40%, NPM1 Exon11 c.860_863dupTCTG p.W288Cfs*12 50%	hydroxycarbamide, IA	46, XY[20]	n/a	n/a
10	AML-M2a	CEBPA p.N356Kfs*65 (Exon1, 48.1%); NPM1 p.W288Cfs*12 (Exon11, 50%); SH2B3 p.A536T (Exon8, 50.8%); RBBP6 p.R1497 (Exon18; 52.7%); GATA2 p.R362Q Exon6, 42.4%); FGFR3 p.L164V (Exon5, 48.5%)	DA- AHAG	46, XX[2]	NR	n/a
19	AML-M2a	NPM1; DNMT3A-ZNF-p.V567del; IDH2-R140Q	CAG	n/a	n/a	n/a
20	AML-M2	NPM1; RB1; TET2; DNMT3A;	MAC	n/a	n/a	n/a

L114	M3	NPM1wt	n/a	n/a	n/a	102299
L1266	M1	NPM1wt	n/a	n/a	n/a	P100751
L1576	M2a	NPM1wt	n/a	n/a	n/a	P148258
L1609	M5	NPM1wt	n/a	n/a	n/a	177360
L1632	M2a	NPM1wt	n/a	n/a	n/a	P156014
L1634	M2a	NPM1wt	n/a	n/a	n/a	P156091
L170	M1	NPM1wt	n/a	n/a	n/a	P035136
L18	M4	NPM1wt	n/a	n/a	n/a	P027819
L2333	M2	NPM1wt	n/a	n/a	n/a	P269018
L31	M1	NPM1wt	n/a	n/a	n/a	100039
L3730	AML-M2a	NPM1wt	n/a	n/a	n/a	D822861
3	n/a	NPM1mu	n/a	n/a	n/a	n/a
4	n/a	NPM1mu	n/a	n/a	n/a	n/a
7	n/a	NPM1mu	n/a	n/a	n/a	n/a
8	n/a	NPM1mu	n/a	n/a	n/a	n/a
9	n/a	NPM1mu	n/a	n/a	n/a	n/a
10	n/a	NPM1mu	n/a	n/a	n/a	n/a
14	n/a	NPM1mu	n/a	n/a	n/a	n/a
20	n/a	NPM1mu	n/a	n/a	n/a	n/a
24	n/a	NPM1mu	n/a	n/a	n/a	n/a
26	n/a	NPM1mu	n/a	n/a	n/a	n/a
30	n/a	NPM1mu	n/a	n/a	n/a	n/a
31	n/a	NPM1mu	n/a	n/a	n/a	n/a
L1251	M5b	NPM1mu	n/a	n/a	n/a	P97359
L1283	M1	NPM1mu	n/a	n/a	n/a	P104164
L1286	M4b	NPM1mu	n/a	n/a	n/a	P105118
L1407	M1	NPM1mu	n/a	n/a	n/a	163116
L4471	AML-M2	NPM1mu	n/a	n/a	n/a	325907
L989	M3	NPM1mu	n/a	n/a	n/a	131943

**Patient samples used for PDX transplantation**						
Sample#	Diagnosis	Mutation	Previous Treatment	Cytogenetics	Response	*HOXBLINC* level
1315# BM	AML	NPM1c+; FLT3 wt	7+3	46,XY[20]	CR	High
921# BM	AML (40% blast)	NPM1c+; FLT3 TKD+	N/A	Normal	N/A	High
LPP4# BM	AML (95% blast)	MLLr+; FLT3wt; NPM1wt	N/A	N/A	N/A	Low

Supplementary Table 1. Human AML patient information

The table lists information of AML patient samples analyzed in this study. Yellow backgrounded samples which were collected at the Institute of Hematology and Blood Disease Hospital, Tianjin, China were used for *HOXBLINC* gene expression analysis. These patients includes 29/65 female, 24/65 male, and 12/65 without sex information. Green backgrounded samplles which were collected by the Pennsylvania State University College of Medicine were used for PDX transplantation. (CR: Complete Remission; PR: Partial Remission; NR: No Response; n/a: information not collected)

Supplementary Table 2 Gene list of common regulated genes by transgenic HoxBlinc and Npm1c+ KI based on RNA-seq

Overlap-HoxBlinc vs NPM1c_VS_WT_UP				
Cldn15	Apom	**Hoxa9**	Slc36a2	Abcc2
NrCAM	Glt1d1	Nupr1	Podxl	Cldn13
Apol11b	Ccdc109b	Tal1	Gm4951	Gm5544
AK171153	Cdr2	Gpr141	Car1	AK005678
Cxcl10	Gdf3	Lrrc25	Serpine2	Tspan15
Gm1604b	Fos	**Hoxb5**	Tnnc2	Epor
Lmo1	Fgfr2	Pi16	Celsr3	Ank1
Ly6i	Mtus1	Wnt5a	Irg1	Plxdc1
Igf1	**Hoxb4**	Met	Pdia2	Mmp19
Kcnq3	Pawr	Lcn2	Tspo2	Ccne1
Tex14	Prss16	Dgat2	AK163054	Homer2
Igsf6	Ednra	Col5a2	Gm14005	Atp7b
Wnt6	Stat1	Bglap	Add2	BC152372
Mir10a	Ptpru	Tubb3	Hpn	Susd2
Selenbp2	**Hoxb2**	Gm6724	Fbn1	C1qc
Gm9733	Mlf1	Sparc	Zfp105	Hist1h3f
C3	Clec4e	Gm16603	Klf1	Apol9b
Hp	Aldh3b1	Cxcl9	Baff	Slc25a21
Slpi	Enah	Mt2	Tns4	Sphk1
Slc2a6	**Hoxa10**	Hmg1l1	Fcgr4	Rbp4
Cyr61	**Runx1**	Sfrp4	Pdgfra	Hist1h2bb
Batf2	Irf8	Zfp534	Kcng2	Slc38a5
AK051451	Glrp1	Cp	Ppbp	Cdx2
Rps6kl1	F13a1	Adamts20	Gm4841	Sh2d4a
1200002N14Rik	Mgst2	H2-Eb1	Ctsg	Mpo
Pik3r6	Lrg1	Atp1b2	Igtp	Gata6
Ly6c2	Cybb	Akap2	Paqr9	Fam55b
Bmx	Guca1a	H2-Ab1	Abcg4	Apba1
Mefv	Galnt3	Acsl6	H60b	Papss2
Wnt5b	**Hoxb3**	Mgam	Gm12250	Dkk4
Cxcr2	Ccno	Tmem56	Epdr1	AK170106
Cebpe	Osm	Ms4a3	AI427809	Irgm1
Kcnj2	Bcat1	Mt1	Anxa3	Zbtb46
Mc1r	Ptgdr	Elane	Ly86	F630028O10Rik
Mir191	**Meis1**	Cited4	Clca1	Il12rb1
Cebpd	Rhou	Cd74	Slc22a4	Zbp1
Abcd2	Ncam1	Hdc	Pklr	Fam132a
Clec5a	Mblac1	Tmod4	Ermap	Gbp2
Gpr150	Nlrp3	Rhag	9130014G24Rik	Rnf207
Ifi47				

Overlap-HoxBlinc vs NPM1c_VS_WT_Down			
Pls3	Tert	Lax1	Pard6b
Vmn2r29	Gpx7	Coro2b	Etv3
Megf11	Bco2	Nhedc2	Coq10b
Gstt1	Plag1	Tmem121	Cbx6
Fgd5	Gm106	Mxra8	Mir678
Cd79a	Cyp2r1	Fscn1	Fam49a
Hspa12b	Pde1c	Mamdc2	Amigo2
St6galnac2	Prkaa2	Tmem44	Igf2r
Gimap7	Rasal1	Il17rc	Prkch
Phyhd1	Akr1c12	Dusp22	Fam102a
Fam115a	Ttll7	Ptn	Fam110b
Ryr1	Rbp1	Myo5c	Axl
Btg3	Gpr162	Bend7	Gm6635
Pvrl3	Hes5	Pgr	DQ548994
Sdc2	Pnck	Gprin3	Ggt6
Fst	Cd28	Clmn	Ccdc157

Tctn2	Prex2	Tnnt3	Pisd-ps1
Ifitm1	Xcl1	Evc	Ptrf
Gprc5a	Ildr1	Cdcp1	Ptms
Gimap6	Aim1l	Npdc1	Amigo1
Adamts12	Gm973	ADAM22	Nlrc3
Dnahc1	Tox	Neil2	Appl2
Gpr161	Rora	Clip4	Nrgn
Cx3cl1	Gdf11	Setbp1	Dbc2
Gimap5	Ctsw	Dsg2	Arhgef11
Prf1	Capsl	Klhl4	Ghr
Epb4.1l4a	Crispld1	Ccdc136	Dab2ip
Vipr2	Rgs11	Samsn1	Fzd3
Cd94	Ltbp4	Avl9	Map4k2
Aipl1	Gch1		

Supplementary Table 3 List of common genes with gain or loss of promoter accessibilities caused by transgenic HoxBlinc and Npm1c+ KI based on ATAC-seq data

overlap.HoxBlinc.VS.NPM1c_VS_WT_ATAC_UP

Fos	Tspan15	Abcc2	Lrrc4c	Zhx1	Aph1b	Uap1
Tex14	Igtp	Wnt5b	4933422A05Rik	Khsrp	Katnb1	B3galnt2
Ccne1	Ifi47	Cybb	Col19a1	Rab5b	Pdss1	Rhoa
Zbp1	Cited4	Sphk1	Axin2	Mbd3	Vezt	Cblb
Cdr2	Gbp2	Hoxa9	G530011O06Rik	Cr2	Trim55	Swsap1
Pawr	Fam132a	Hdc	Pex2	Phf19	Eny2	Pcna
Ccno	Lrrc25	Atp1b2	Esp38	Opa3	Casp8ap2	Gm8579
Ly6c2	Cebpd	Igsf6	Rab10os	Rev3l	Fiz1	Slc35f5
Runx1	Paqr9	Fgfr2	Limd1	Rasa1	Dscr3	Eaf2
Kcnj2	Gm9733	Batf2	Txndc11	Nfya	Grk4	Ppp2r5c
Galnt3	Zbtb46	Abcg4	Chl1	BC003331	Rab8a	Kpna3
Stat1	Mlf1	Rhag	Cdk8	Hook3	Bcl2l1	Birc2
Celsr3	Gm5544	Homer2	Vav1	Wnt7b	Cdkn2aip	Zfp367
Hist1h3f	Ptpru	Irg1	Filip1l	Rps29	Mpc1	Rps19
Fcgr4	Glt1d1	Sfrp4	Satl1	Hic2	Vkorc1l1	Epc2
Apba1	Adamts20	Il12rb1	1700019M22Rik	Pja2	Pcx	Usp22
H60b	Mefv	Akap2	Stat2	Nfyc	2610044O15Rik8	Zfyve16
Met	Slpi	Pik3r6	Dhrsx	Ddx54	Thap1	Srd5a1
Mblac1	Slc2a6	Ctsg	Gphn	Mier3	Sf1	Ccdc71l
Gpr150	Irf8	Pdgfra	Mucl1	Rfx1	Nck1	Scai
Ank1	Lmo1	Acsl6	Lrif1	Carm1	Kdm4b	Zbtb21
Aldh3b1	Podxl	Wnt6	Myl12b	Izumo4	Tom1	Wdr89
Susd2	Hoxb5	Irgm1	Ppp2r5a	Mcph1	Pdp1	Mib1
Dgat2	Sh2d4a	Hoxa10	Ppp2r3d	Eif2ak3	Vcp	Ube2g1
Meis1	Epdr1	Igf1	Myom2	Sap130	2610206C17Rik	Rock1
Klf1	Enah	Gm12250	Rap2a	Cetn3	Zfp869	Eif2s3x
Mmp19	Ptgdr	Sparc	Dtnbp1	Tomm70a	Paip2b	Pkn2
Prss16	Glrp1	Mpo	4932412D23Rik	Zfp958	Suco	Bclaf1
Serpine2	Papss2	Bmx	Cic	Eed	Gm7244	Zfp319
Ly6i	Nupr1	Mc1r	Sp4	Azi2	Papss1	AI597479
Gpr141	Tal1	Cxcl10	Ep400	7-Sep	Leng8	Tomm40
Guca1a	Clec5a	Cxcr2	Zfand5	Vapa	Immt	Ap1ar
Plxdc1	Rhou	Cd74	Tmem5	Epm2aip1	Mastl	Golga5
Ncam1	Osm	Lrg1	Rab40c	Zzz3	Hyls1	Ptprs
Ly86	Ms4a3	Mt2	Mir7021	Tmem29	Ubn2	Rbm15
Gm14005	H2-Ab1	Cp	Crem	Tmtc4	Myo9a	Hnrnpc
Mgst2	Anxa3	Tubb3	Crocc	Atp2b1	Usp20	
Fbn1	Slc25a21	Cldn13	Pdcd10	Malt1	Mtrf1l	
Bcat1	Tns4	Rps6kl1	Dnpep	Lrig2	Fbxl4	
Ccdc109b	Clec4e	Hpn	Mon1b	Rab5a	Cyth2	
Tmem56	Hoxb2	Cdx2	1190002N15Rik	Fgfr1op	Ttc14	
Cebpe	Nlrp3	Mgam	Sp1	Mfsd8	Sacs	
Abcd2	Epor	C1qc	Limk1	Map3k3	Phtf2	
Mt1	Kcng2	Atp7b	9430038I01Rik	Slc25a42	Zfp619	
AI427809	Ednra	Mir10a	Nbn	Dtwd2	Nbeal1	
F13a1	Hoxb3	C3	Sypl	Hsd17b12	Erp44	
Mtus1	Hp	Slc36a2	Pole4	Bbip1	Mcm4	
Slc22a4	Pi16	Zfp105	Smarca5	Calm3	Upf3a	
Cyr61	Kcnq3	Zfp534	Mir5620	Baz1b	Tusc1	

overlap.HoxBlinc.VS.NPM1c_VS_WT_ATAC_down

Avl9	Ptrf	Rbp1	Dbc2	4921509O07Rik	Txndc16
Coq10b	Ggt6	Ptn	2810459M11Rik	Tmem51	Nostrin
Pisd-ps1	Clmn	Capsl	Rnd3	1700042G07Rik	Tlr8
Vmn2r29	Sdc2	Ryr1	Onecut1	Pex16	BC023829
Fam110b	Etv3	Neil2	E330017L17Rik	Dock3	Thoc6
Gprc5a	Cx3cl1	Pnck	Ppp2r4	Soat2	Apln
Gch1	Il17rc	Tert	H2-Ea-ps	Gm6116	Sephs2
Pard6b	Igf2r	Myo5c	Slfn5	Gm14718	Npl
Fam49a	Tmem121	Ltbp4	Gm13139	6030466F02Rik	Fam129a
Fam102a	Phyhd1	Ifitm1	Clu	Samd5	Scimp
Prkch	Rasal1	Gpr162	Mrpl16	Gm11548	Slx4ip

Bend7	Clip4	Fst	Stpg1	Prg2	Macf1
Aipl1	Gpx7	Xcl1	E330021D16Rik	Fam129b	Extl1
Epb4.1l4a	Appl2	Megf11	5430434I15Rik	Fam124a	Mycbpap
Ttll7	Mamdc2	St6galnac2	Pkdrej	Ccdc63	Cacng1
Amigo2	Cd28	Akr1c12	Bpi	Cd59a	Fam169a
Fzd3	Axl	Klhl4	Tmem173	Oit3	Saal1
Pvrl3	Gstt1	Crispld1	Srgn	Olfm2	Lrmp
Gprin3	Npdc1	Rgs11	Mir32	Zscan10	Gm15800
Prkaa2	Hspa12b	Adamts12	Msantd1	Abcb5	Chrnb3
Cyp2r1	Plag1	Ctsw	Tspan4	Baiap2l2	Pald1
Tmem44	Prf1	Dsg2	Nkd2	Mroh5	Cd99l2
Setbp1	Nlrc3	Pde1c	Knop1	Dnajc22	
Ghr	Bco2	Cdcp1	Pnp2	Vmn2r88	
Gimap6	Coro2b	Evc	Decr2	Klhl29	
Pgr	Hes5	Cd79a	Rnf217	Mllt6	
Dab2ip	Pls3	Gimap7	Arhgef9	Nckap5	
Fgd5	Mxra8	Dnahc1	Ankrd29	5830473C10Rik	
Vipr2	Fscn1	Cd94	Elk3	Klhl1	
Arhgef11	Prex2	Gm106	Srpk1	Cars2	
Gpr161	Gm973	Aim1l	Ofcc1	Slc26a3	
Map4k2	Nrgn	Nhedc2	Dclk3	Mir6897	
Ptms	Btg3	Tnnt3	Ubash3a	Trp73	
Fam115a	Gdf11	ADAM22	Vps33b	Tgm5	
Samsn1	Txnip	Cbx6	Ssr2	Foxi3	
Ildr1	Tctn2	Mir678	Dll1	Dagla	
Gimap5	Lax1	Gm6635	Usp35	Ccdc12	
Tox	Ccdc136	DQ548994	Slc44a2	Tmem150b	
Amigo1	Rora	Ccdc157	Zfp710	Mafg	

Supplementary Table 4 List of HoxBlinc ChIRP transcription motif analysis

WT			Tg	
**Motif**	**p-Value**		**Motif**	**p-Value**
ETV1	1.00E-325		E-box	1.00E-280
USF1	1.00E-250		CRE	1.00E-260
E-box	1.00E-232		CTCF	1.00E-228
Elf4	1.00E-201		PU.1	1.00E-201
Gata1	1.00E-179		ETS1	1.00E-181
STAT3	1.00E-152		c-Myc	1.00E-136
CRE	1.00E-132		USF1	1.00E-109
CTCF	1.00E-104		**STAT1**	1.00E-92
YY1	1.00E-92		ATF1	1.00E-88
Max	1.00E-65		AP1	1.00E-80
TEAD1	1.00E-52		STAT2	1.00E-77
USF1	1.00E-42		GATA2	1.00E-69
STAT1	1.00E-35		Nanog	1.00E-54
PU.1	1.00E-30		STAT3	1.00E-44
GATA2	1.00E-26		TEAD1	1.00E-38
ETS1	1.00E-22		ERRA	1.00E-29
STAT2	1.00E-14		Bcl11a	1.00E-21
ELK4	1.00E-11		Elf4	1.00E-15
Bcl11a	1.00E-09		Max	1.00E-12
ELF1	1.00E-08		TCF12	1.00E-10
ATF1	1.00E-07		USF2	1.00E-08
ERRA	1.00E-07		MITF	1.00E-07
c-Myc	1.00E-06		TCF4	1.00E-07
Nanog	1.00E-06		ERG	1.00E-06
MITF	1.00E-05		TBX5	1.00E-05
ERG	1.00E-05		Smad3	1.00E-05

p -value was calculated by two-tailed student's t test.

Supplementary Table 5 List of common genes of HoxBlinc ChIRP-seq, H3K4me3 ChIP-seq, and MLL1 ChIP-seq

Overlap_H3K4_chirp_MLL_CHIP_up_gain
Wnt5b
**Runx1**
Slc38a5
**Meis1**
Xcr1
Pros1
Nup35
Casp1
Hdhd3
Lmo1
**Hoxa9**
Tcf7l1
Abcg4
Hmbs
Fam55b
Rexo2
**Hoxb4**
**Hoxb2**
Ncam1
Alas1
Manf
Cdx2
**Hoxb5**
**Cdr2**
Cacna1f
**Hoxa10**
Mir10a
LOC100270707
Rnf128
Wnt5a
Cysltr1
Mir760
Met
Irf8
**Stat1**
Il13ra1
Gpc3
Mpp1
Mir486

Supplementary Table 6 Sequences of primers, shRNAs and sgRNAs

Primers' name	Strand	Sequence (sequence 5' to 3')
**Genotype primers:**		
HoxBlinc^{Tg} genotyping P1	F	GGGCGACAGTTACAGTCACAGA
HoxBlinc^{Tg} genotyping P2	R	CTAACCCCAATGCCCTCCTA
**RT-qPCR primers:**		
hHoxB2	F	TCCTCCTTTCGAGCAAACCTTCC
hHoxB2	R	AGTGGAATTCCTTCTCCAGTTCC
hHoxB3	F	AGTACAAGAAGGACCAGAAGGC
hHoxB3	R	TGGAGTGTAAGGCGTTCATG
hHoxb4	F	TTCACGTGAGCACGGTAAAC
hHoxb4	R	TTCCTTCTCCAGCTCCAAGA
hHoxB5	F	GTTCCACTTCAACCGCTACC
hHoxB5	R	TGTCCTTCTTCCACTTCATGC
hHOXB6	F	GGCGAGGCCGCCAGACATAC
hHOXB6	R	ACTCGGCCTGTTTTTCTTCC
hHoxB7	F	AGAGTAACTTCCGGATCTA
hHoxB7	R	TCTGCTTCAGCCCTGTCTT
hHoxB9	F	GAGCAGGGCAAAGAGTAA
hHoxB9	R	CTTTCTCCTGACACCTAG
hHOXB13	F	CTGGAACAGCCAGATGTGTT
hHOXB13	R	TTGGCGAGAACCTTCTTCTC
hHoxBlinc-set1	F	TGAGCAGATTTCCTTATC
hHoxBlinc-set1	R	CTCCTACTTACTGTCAAG
hHoxBlinc-set2	F	GGTTGGATGAAGAGAAGA
hHoxBlinc-set2	R	GAGCAGGCAGTTACTATA
mHoxBlinc-set1	F	ATCTCAGGTTCCAGCTTCACTC
mHoxBlinc-set1	R	GGCTTCCTCAATCACTTCCA
mHoxBlinc-set2	F	ATTGCCCGTCATTAAATATG
mHoxBlinc-set2	R	GTGTGAAGGGGGTGTAAT
mHoxB1	F	CCATATCCTCCGCCGCAG
mHoxB1	R	CGGACTGGTCAGAGGCATC
mHoxB2	F	GCTCGCCGAGTGTCTGACTT
mHoxB2	R	AATGTCGACTCCTTGATTGATGAA
mHoxB3	F	TACCAGCGCTCAGCGTGTT
mHoxB3	R	TGCCATTGAGCTCCTTGCT
mHoxB4	F	TGGATGCGCAAAGTTCACG
mHoxB4	R	GGTCTTTTTTCCACTTCATGCG
mHoxB5	F	TCCTCTGAGCCCGAGGAAGCGGCGAG
mHoxB5	R	CCACTTCATGCGACGGTTCTG
mHoxB6	F	TTCCTATTTCGTGAACTCCACCTT
mHoxB6	R	CCGCATAGCCAGACGAGTAGA
mHoxB7	F	ACCGAGTTCCTTCAACATGC
mHoxB7	R	CCGAGTCAGGTAGCGATTGT
mHoxB8	F	GACTCGCAAGCGGAGGATC
mHoxB8	R	GAACCAGATTTTGACCTGTCTCTCT
mHoxB9	F	TGTCCATTTCTGGGACGCTTA
mHoxB9	R	GAACACCGGCGCTTTGG
mHoxB13	F	CTGGAACAGCCAGATGTGTT
mHoxB13	R	CCTGCTAAAGGTGTCATCTC
mMeis1	F	ATGACACGGCATCCACTCG
mMeis1	R	TTTTTGTCCTTATCAGGGTCAT
mRunx1	F	GAGGTGCGTTTTCGAAAGGA
mRunx1	R	CTCATCTTGCCGGGGCTCAG
**4C primers:**		
4C-B4-bait	F	AATGATACGGCGACCACCGAGATCTACACTCTTTCCCTACACGACGCTCTTCCGATCT GGCAAGTGTGATGGAAAAATACC

4C-B4-bait-WT	R	CAAGCAGAAGACGGCATACGAGATGTCCGCGTGACTGGAGTTCAGACGTGTGCTCTT CCGATCGCGGCTGGGTAGGTTTTAAT
4C-B4-bait-HoxBlincTg	R	CAAGCAGAAGACGGCATACGAGATCCGTCCGTGACTGGAGTTCAGACGTGTGCTCTT CCGATC GCGGCTGGGTAGGTTTTAAT
4C-43Kb-bait	F	AATGATACGGCGACCACCGAGATCTACACTCTTTCCCTACACGACGCTCTTCCGATCT AGCTCCTGGGCACTGATTC
4C-43Kb-bait-WT	R	CAAGCAGAAGACGGCATACGAGATCAGATCGTGACTGGAGTTCAGACGTGTGCTCTT CCGATC CTTTCCCCTCCTATGCCTGT
4C-43Kb-bait-HoxBlincTg	R	CAAGCAGAAGACGGCATACGAGATACAGTGGTGACTGGAGTTCAGACGTGTGCTCTT CCGATC CTTTCCCCTCCTATGCCTGT
4C-B13-bait	F	AATGATACGGCGACCACCGAGATCTACACTCTTTCCCTACACGACGCTCTTCCGATCT TGTTCCTCGGGGCTATCCT
4C-B13-bait-WT	R	CAAGCAGAAGACGGCATACGAGATCTTGTAGTGACTGGAGTTCAGACGTGTGCTCTTC CGATCTTCAGAGAGGCTGGTGGCT
4C-B13-bait-HoxBlincTg	R	CAAGCAGAAGACGGCATACGAGATGGCTACGTGACTGGAGTTCAGACGTGTGCTCTT CCGATCTTCAGAGAGGCTGGTGGCT
4C-73Kb-bait	F	AATGATACGGCGACCACCGAGATCTACACTCTTTCCCTACACGACGCTCTTCCGATCT GGCTGGCCTTGAATTCACA
4C-73Kb-bait-WT	R	CAAGCAGAAGACGGCATACGAGATAGTCAAGTGACTGGAGTTCAGACGTGTGCTCTTC CGATCTCTTTTGGGAGCTGAGGCA
4C-73Kb-bait-HoxBlincTg	R	CAAGCAGAAGACGGCATACGAGATGCCAATGTGACTGGAGTTCAGACGTGTGCTCTT CCGATCTCTTTTGGGAGCTGAGGCA
**ATAC primers:**		
Ad1		AATGATACGGCGACCACCGAGATCTACACTCGTCGGCAGCGTCAGATGTG
Ad2.2_CGTACTAG		CAAGCAGAAGACGGCATACGAGATCTAGTACGGTCTCGTGGGCTCGGAGATGT
Ad2.3_AGGCAGAA		CAAGCAGAAGACGGCATACGAGATTTCTGCCTGTCTCGTGGGCTCGGAGATGT
Ad2.4_TCCTGAGC		CAAGCAGAAGACGGCATACGAGATGCTCAGGAGTCTCGTGGGCTCGGAGATGT
Ad2.5_GGACTCCT		CAAGCAGAAGACGGCATACGAGATAGGAGTCCGTCTCGTGGGCTCGGAGATGT
Ad2.6_TAGGCATG		CAAGCAGAAGACGGCATACGAGATCATGCCTAGTCTCGTGGGCTCGGAGATGT
Ad2.7_CTCTCTAC		CAAGCAGAAGACGGCATACGAGATGTAGAGAGGTCTCGTGGGCTCGGAGATGT
Ad2.8_CAGAGAGG		CAAGCAGAAGACGGCATACGAGATCCTCTCTGGTCTCGTGGGCTCGGAGATGT
**ChIRP-PCR primers**		
Runx1	F	GAAAGCCTGTGGTTTGCATT
Runx1	R	AGCCTGGCAGTGTCAGAAGT
Hoxb1	F	ACTGCCGAAAGGTTGTAG
Hoxb1	R	CATCCTATTATAGTCCATGTAGAG
Hoxb2	F	ACTAGCGATTGGCGGAGAC
Hoxb2	R	ATAAATACAAGCGTATGGGGACTC
Hoxb3	F	AAACAGCCTCCCCTTTGAAT
Hoxb3	R	TGTGCATCTGCTGGAATCTC
Hoxb4	F	GCCTCTAACTTTGTTCACTTGAC
Hoxb4	R	AGCCATTAATTTCTGGGAATTGC
Hoxb5	F	GAGAATTTACGACTGGTCAAC
Hoxb5	R	ATAGCGATGCACTGTACTTC
Hoxb6	F	TCCCGATGAGTTCCTATTTC
Hoxb6	R	GCATAGCCAGACGAGTAG
Hoxb8	F	GGGGTCTCTAATGGATGCAA
Hoxb8	R	GAAGAAGGGATGCCAGTTCA
Hoxb9	F	TGGATATGGAATGCGGTGAG
Hoxb9	R	TGCGGACTGCCTGATAAAG
**ChIRP probes**		**sequence**
HoxBlinc probe1		TTGACCCAAGCATCAAGCAA
HoxBlinc probe2		TAGGCCAGAGGCATACTTAA
HoxBlinc probe3		ACCTCATAGATCTAGGCATG
HoxBlinc probe4		CTTGCAAATGGGCAGGAGAG
HoxBlinc probe5		TCTGCCCTTACTGAGTAAAT
HoxBlinc probe6		GTTTAAACTGGGAGAGCTGT
HoxBlinc probe7		TGTGTGGCCAAAATGGGATT
HoxBlinc probe8		AGGCTGGAATAAGGGGACAC
HoxBlinc probe9		ATTCCGTTTACAACCTGAGA
HoxBlinc probe10		GGTGTGAAGGGGGTGTAATA
HoxBlinc probe11		gcatcaagcaagaacagtcc
HoxBlinc probe12		tctgcccttactgagtaaat

HoxBlinc probe13		tcaaaggcagttcatttccg
HoxBlinc probe14		ggtgtgaagggggtgtaata
HoxBlinc probe15		tgcctcgaagtcagaatcag
HoxBlinc probe16		gttgtgcaaagggactcgaa
HoxBlinc probe17		agacagacaggtcttcttgg
HoxBlinc probe18		atgagcggagtgtttatgtc
HoxBlinc probe19		acacctcaggtagatgagat
HoxBlinc probe20		ccaacatcacacgcacaaga
HoxBlinc probe21		tccagattcgtcgaagcaat
HoxBlinc probe22		catttagctgcactatgagc
HoxBlinc probe23		cagagggacttgtcagagat
HoxBlinc probe24		ttcttgaaatgggtacccac
HoxBlinc probe25		tgtggggttttaagtggttg
LacZ probe1		CAGTTGGTCTGGTGTCAAAA
LacZ probe2		TAGGGCCGCAAGAAAACTAT
LacZ probe3		ACGACATTGGCGTAAGTGAA
LacZ probe4		ATTGAACTGCCTGAACTACC
LacZ probe5		GTGGATCAGTCGCTGATTAA
LacZ probe6		GTGGATCAGTCGCTGATTAA
LacZ probe7		TTGAAAATGGTCTGCTGCTG
LacZ probe8		GCCACTCGCTTTAATGATGA
LacZ probe9		GGTTGTTACTCGCTCACATT
LacZ probe10		TTTTACAACGTCGTGACTGG
**sgRNA primers**		**sequence**
HoxBlinc- KRAB-F		GGAAGACTTGTGGGTTGCAG
HoxBlinc- KRAB-R		CTGCAACCCACAAGTCTTCC
**TAIL-PCR-Primers**		**Sequence**
Arbitrary Degenerate (AD)		NTCGASTWTSGWGTT
Specific primers (Sp)-1		TGTGCACAGAGTCCTGGGTA
Specific primers (Sp)-2		TCCTGGGTACCCGGGGAT
Specific primers (Sp)-3		AGTCGAGCTCGCGAAAGCT
**shRNA sequence**		**Sequence**
sh*Mll1*-1# (Sigma		CCGGACGAAGATGACTTATACTATTCTCGAGAATAGTATAAGTCATCTTCGTTTTTTG
sh*Mll1*-2# (Sigma		CCGGGGTCCCTAGTGTCCTACATATCTCGAGATATGTAGGACACTAGGGACCTTTTTG
sh*Setd1a*-1#		TGCTGTTGACAGTGAGCGCCAAGTTCCCACTAGAAGACAATAGTGAAGCCACAGATGT ATTGTCTTCTAGTGGGAACTTGTTGCCTACTGCCTCGGA
sh*Setd1a*-2#		TGCTGTTGACAGTGAGCGCTAGCAAGAAGGAGAAAGATAATAGTGAAGCCACAGATGT ATTATCTTTCTCCTTCTTGCTAATGCCTACTGCCTCGGA
shHOXBLINC-1#		CCATCCAGCTGCAGAGAAA
shHOXBLINC-2#		GGAATTGCCCGTCATTAAA

Supplementary Table 7 List of experiments and data analysis resources

REAGENT OR RESOURCE	SOURCE	IDENTIFIER
**Antibodies**		
Anti-H3K4me3 antibody, rabbit monoclonal	Millipore	04-745; RRID:AB_1163444
Anti-mouse CD34 Alexa Fluor 700	eBioscience	56-0341-82; RRID: AB_493998
Rat anti-Mouse Ly-6A/E (Sca1) PE-Cy7	BD Pharmingen	558162; RRID: AB_647253
Rat anti-Mouse CD117 PE	BD Pharmingen	553355; RRID: AB_394806
Rat anti-Mouse CD117 APC	BD Pharmingen	553356; RRID: AB_398536
Rat anti-Mouse CD117 PerCP-Cy5.5	BD Pharmingen	560557; RRID: AB_1645258
Rat anti-Mouse CD117 FITC	BD Pharmingen	553354; RRID: AB_394805
Rat anti-Mouse CD16/32 APC-Cy7	BD Pharmingen	560541; RRID: AB_1645229
Rat anti-mouse CD135 BV421	BD Horizon	562898; RRID: N/A
Mouse Lineage Antibody Cocktail APC	BD Pharmingen	51-9003632; RRID: N/A
Rat anti-Mouse CD71 FITC	BD Pharmingen	553266; RRID: AB_394743
Rat anti-Mouse CD71 PE	BD Pharmingen	553267; RRID: AB_394744
Rat anti-Mouse TER-119 APC	BD Pharmingen	557909; RRID: AB_398635
Anti-Mouse CD41 APC	eBioscience	17-0411-80; RRID: AB_1603238
Anti-Mouse/Rat CD61 PE	eBioscience	12-0611-81; RRID: AB_465717
Rat anti-Mouse Ly-6G and Ly-6C PerCP-Cy5.5	BD Pharmingen	552093; RRID: AB_394334
Rat anti-Mouse Ly-6G and Ly-6C PE-Cy7	BD Pharmingen	552985; RRID: AB_394535
Rat anti-Mouse CD11b PE	BD Pharmingen	553311; RRID: AB_394775
Rat anti-Mouse IgM FITC	BD Pharmingen	553437; RRID: AB_394857
Rat anti-Mouse CD45R/B220 APC	BD Pharmingen	553092; RRID: AB_398531
Rat anti-Mouse CD45R/B220 PE	BD Pharmingen	553090; RRID: AB_394620

Rat anti-Mouse CD4 PE-Cy7	BD Pharmingen	552775; RRID: AB_394461
Rat anti-Mouse CD4 FITC	BD Pharmingen	553729; RRID: AB_395013
Rat anti-Mouse CD8a PE	BD Pharmingen	553033; RRID: AB_394571
Mouse anti-Mouse CD45.2 PerCP-Cy5.5	BD Pharmingen	552950; RRID: AB_394528
Mouse anti-Mouse CD45.1 FITC	BD Pharmingen	553775; RRID: AB_395043
Mouse Anti-Human CD45 APC	BD Pharmingen	555485; RRID: AB_398600
Mouse Anti-Human CD3 BV421	BD Pharmingen	562426; RRID: AB_ 11152082
Mouse Anti-Human CD33 PE	BD Pharmingen	561816; RRID: AB_10896480
Mouse Anti-Human CD19 FITC	BD Pharmingen	560994; RRID: AB_10563406
Mouse Anti-Human CD45 PerCP-Cy5.5	BD Pharmingen	564105; RRID: AB_2744405
Mouse Anti-Human CD34 APC	BD Pharmingen	560940; RRID: AB_10563908
Human Myeloperoxidase/MPO Antibody (for IHC)	R&D	MAB3174; RRID: AB_2250873
Anti-CD45 antibody (for IHC)	Abcam	ab10559; RRID: AB_442811
Biotin Goat Anti-Rabbit IgG	BD Pharmingen	550338; RRID: AB_393618
Biotin Rat Anti-Mouse IgG2b	BD Pharmingen	550333; RRID: AB_393613
Anti-KMT2A/MLL1 antibody, rabbit polyclonal	Novus Biologicals	NB600-248, RRID: AB_2145479
Anti- LSD1/BHC110 antibody, rabbit polyclonal	Millipore	07-705, RRID: AB_441950
Anti- hSET1 Antibody, rabbit polyclonal	Bethyl	A300-289A, RRID: AB_263413
**Chemicals, enzymes**		
Lipofectamine 3000 reagent	Thermo Fisher Scientific	L3000-008
Proteinase K	Thermo Fisher Scientific	25530049
Protease inhibitor Cocktail	Abcam	ab65621

Puromycin	Thermo Fisher Scientific	A1113802
Dynabeads™ Protein G	Thermo Fisher Scientific	10003D
Dynabeads™ Protein A	Thermo Fisher Scientific	10001D
AMPure XP	Beckman Coulter	A63881
**Experimental Models: Cell lines**		
HEK293T	ATCC	CRL-3216
AML cell lines see Table S8		
**Experimental Models: Organisms/Strains**		
*HoxBlinc*transgenic mice	Mingjiang Xu lab, UT Health SA	N/A
NOD-*scid* IL2Rγ^{null}mice	The Jackson Laboratory	005557
B6.SJL-Ptprca Pepcb/BoyJ mice	The Jackson Laboratory	002014
**Plasmids**		
HS321/45-vav vector	Mingjiang Xu lab, UT Health SA	N/A
pTRIPz	Horizon Discovery	
pGIPZ	Horizon Discovery	
hPGK-Puro-CMV-tGFP	Sigma	
pMD2.G	Addgene	Plasmid # 12259
psPAX2	Addgene	Plasmid # 12260
pGEM®-T Easy Vector Systems	Promega	A137A
**Critical Commercial Assays**		
RNeasy mini-isolation kit	QIAGEN	74106
QIAquick Gel Extract kit	QIAGEN	28706
Superscript II reverse Transcriptase	Thermo Fisher Scientific	18064014
QIAuick PCR purification kit	QIAGEN	28106
QIAprep Spin Miniprep Kit	QIAGEN	27106

QIAGEN Plasmid *Plus* Maxi Kit	QIAGEN	12965
Nextera DNA Library Preparation Kit	Illumina	FC-121-1030
**Deposited Data**		
CHIP-SEQ in *HOXBLINC* WT/ Transgenic cells	This study	GEO:GSE115096
RNA-SEQ in *HOXBLINC* WT/ Transgenic mice	This study	GEO:GSE115096
4C-SEQ in *HOXBLINC* WT/ Transgenic mice	This study	GEO:GSE115096
ATAC-SEQ in *HOXBLINC* WT/ Transgenic mice	This study	GEO:GSE115096
**Oligonucleotides**		
Genotype primers	This stydy	see Table S6
RT-qPCR primers	This study	see Table S6
ATAC&4C primers	This study	see Table S6
**Software and Algorithms**		
TopHat/2.0.13	(Trapnell et al., 2012)	https://ccb.jhu.edu/software/tophat/
GraphPad Prism 8.0	Graphpad, Inc	https://www.graphpad.com/scientific-software/prism/
Bowtie2/2.2.9	(Langmead and Salzberg, 2012)	http://bowtiebio.sourceforge.net/bowtie2/
R/3.6.1	NA	https://www.r-project.org/
Cufflinks/2.2.1	(Trapnell et al., 2010)	http://cole-trapnell-lab.github.io/cufflinks/
Cuffdiff/2.2.1	(Trapnell et al., 2010)	http://cole-trapnell-lab.github.io/cufflinks/
Integrated Genomic Viewer 2.4.19	(Robinson et al., 2011)	http://software.broadinstitute.org/
Deeptools/3.1.3	(Ramirez et al., 2014)	https://deeptools.github.io/
Gene Set Enrichment Analysis (GSEA) 4.0.0	(Subramanian et al., 2005)	http://software.broadinstitute.org/gsea/

Supplementary Table 8 Detailed information of AML cell lines

Cell line name	Disease	Sequence variations			
K562	CML	BCR-ABL1 fusion	Homozygous for TP53 p.Gln136fs*13		
MOLM-13	AML	MLL-AF9 fusion	FLT3-ITD		
MV4-11	AML	MLL-AFF1 fusion	FLT3-ITD		
THP-1	AML	CSNK2A1-DDX39B fusion	MLL-AF9 fusion	Heterozygous for NRAS p.Gly12Asp	us for TP53 p.Arg174fs*3
NOMO1	AML	MLL-AF9 fusion	Heterozygous for EP300 c.730-10T>C	Heterozygous for KRAS p.Gly13Asp	Heterozygous for TP53 p.Cys242fs*5
SET-2	AML	Heterozygous for JAK2 p.Val617Phe			
OCI-AML2	AML	MLL-AF6 fusion			
OCI-AML3	AML	Heterozygous for NPM1 p.Trp288Cysfs*12	DNMT3A p.Arg882Cys		
IMS-M2	AML	Heterozygous for NPM1 p.Trp288Cysfs*12			

Supplementary Table 9. FACS gating strategy

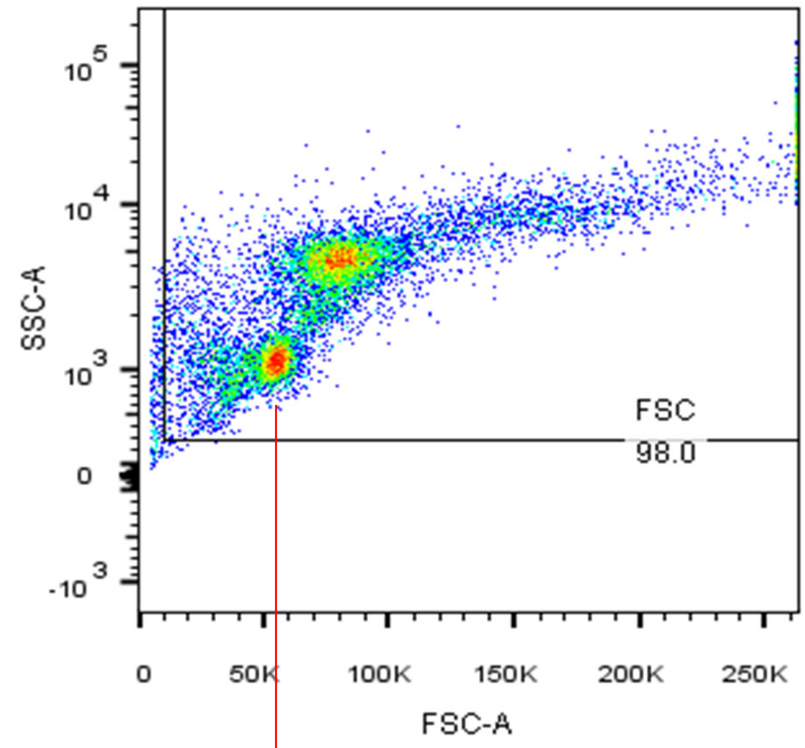

FSC/SSC gate was set to gate all cells in, but exclude small debris.

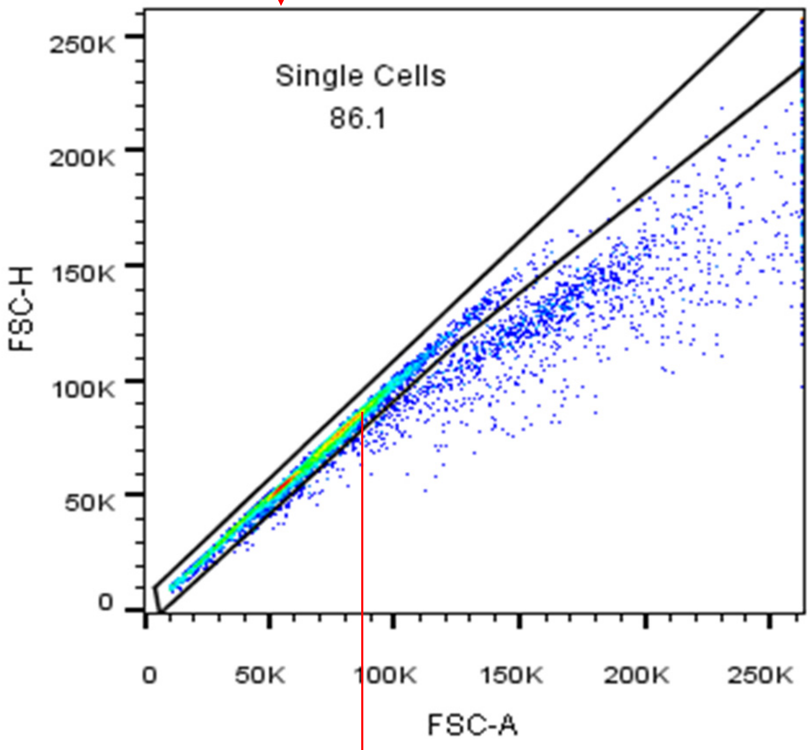

FSC-A/SSC-H gate was set to gate all single cells, but exclude cell clumps.

To all following gate for analysis as shown in Figures.
