# Supplementary material for: HOXBLINC long non-coding RNA activation promotes leukemogenesis in NPM1-mutant acute myeloid leukemia
Source: Nat Commun. 2021 Mar 29;12:1956. doi: 10.1038/s41467-021-22095-2 (PMC8007823; doi:10.1038/s41467-021-22095-2)
Supplement: Supplementary file 3 — Reporting Summary [file 41467_2021_22095_MOESM3_ESM.pdf]

nature research

Corresponding author(s): Mingjiang Xu

Last updated by author(s): Jan 15, 2021

Reporting Summary

Nature Research wishes to improve the reproducibility of the work that we publish. This form provides structure for consistency and transparency in reporting. For further information on Nature Research policies, see our Editorial Policies and the Editorial Policy Checklist.

Statistics

For all statistical analyses, confirm that the following items are present in the figure legend, table legend, main text, or Methods section.

n/a	Confirmed	
☐	☒	The exact sample size (*n*) for each experimental group/condition, given as a discrete number and unit of measurement
☐	☒	A statement on whether measurements were taken from distinct samples or whether the same sample was measured repeatedly
☐	☒	The statistical test(s) used AND whether they are one- or two-sided<br>*Only common tests should be described solely by name; describe more complex techniques in the Methods section.*
☒	☐	A description of all covariates tested
☐	☒	A description of any assumptions or corrections, such as tests of normality and adjustment for multiple comparisons
☐	☒	A full description of the statistical parameters including central tendency (e.g. means) or other basic estimates (e.g. regression coefficient) AND variation (e.g. standard deviation) or associated estimates of uncertainty (e.g. confidence intervals)
☐	☒	For null hypothesis testing, the test statistic (e.g. *F*, *t*, *r*) with confidence intervals, effect sizes, degrees of freedom and *P* value noted<br>*Give P values as exact values whenever suitable.*
☒	☐	For Bayesian analysis, information on the choice of priors and Markov chain Monte Carlo settings
☒	☐	For hierarchical and complex designs, identification of the appropriate level for tests and full reporting of outcomes
☐	☒	Estimates of effect sizes (e.g. Cohen's *d*, Pearson's *r*), indicating how they were calculated

Our web collection on statistics for biologists contains articles on many of the points above.

Software and code

Policy information about availability of computer code

Data collection	BD FACSDIVA™ SOFTWARE Version 8.0 for Windows was used to collect all FACS data.<br>Illumina HiSeq 2500/3000, NextSeq 500 were used for raw sequencing data collection.
Data analysis	FlowJo V10 for Windows was used to analyze all FACS data.<br>TopHat (v. 2.0.13), Bowtie2 (v. 2.2.9), R (v. 3.6.1), Cufflinks (v.2.2.1), Cuffdiff (v.2.2.1), Integrated Genomic Viewer (v. 2.4.19), Deeptools (v. 3.1.3), Gene Set Enrichment Analysis (GSEA)(v.4.0.0) were used for sequencing data analysis.<br>GraphPad Prism 8.0 was used for statistics.<br>Website links of Software and algorithms are provided in Supplementary Table 7.

For manuscripts utilizing custom algorithms or software that are central to the research but not yet described in published literature, software must be made available to editors and reviewers. We strongly encourage code deposition in a community repository (e.g. GitHub). See the Nature Research guidelines for submitting code & software for further information.

Data

Policy information about availability of data

All manuscripts must include a data availability statement. This statement should provide the following information, where applicable:

- Accession codes, unique identifiers, or web links for publicly available datasets
- A list of figures that have associated raw data
- A description of any restrictions on data availability

Sequencing data of RNA-seq, ATAC-seq, ChIP-seq and 4C-seq in Figure 1, 4, 5, 6, S1, S2, S6, S7 and S9 were deposited in the Gene Expression Omnibus (accession number GSE115096, https://www.ncbi.nlm.nih.gov/geo/query/acc.cgi?acc=GSE115096). RNA-seq data of AML patient samples were retrieved from The Cancer Genome Atlas (GEO accession number GSE62944, https://www.ncbi.nlm.nih.gov/geo/query/acc.cgi?acc=GSE62944) for Figure 1 and S1. NCBI GEO public database

(GSM918748, https://www.ncbi.nlm.nih.gov/geo/query/acc.cgi?acc=GSM918748) were used for CTCF binding site analysis of Figure 5 and S7. Source data are provided with this paper. All biological material were either directly commercially available or are available upon request from the lab (see Table S7). Supplemental Information Supplemental information includes 9 figures and 9 tables. All other data are available from the authors.

Field-specific reporting

Please select the one below that is the best fit for your research. If you are not sure, read the appropriate sections before making your selection.

- [x] Life sciences
- [ ] Behavioural & social sciences
- [ ] Ecological, evolutionary & environmental sciences

For a reference copy of the document with all sections, see nature.com/documents/nr-reporting-summary-flat.pdf

Life sciences study design

All studies must disclose on these points even when the disclosure is negative.

Sample size	For in-vitro experiments based on cells, a sample size of n=3 independent experiments was used throughout the study. No sample size calculation was performed beforehand.<br><br>For in-vivo experiments based on mice, group sizes were determined by an a prior power analysis for a two-sided, two-sample t-test with an α of 0.05 and power of 0.9 to detect a 50% difference in parameter between groups.
Data exclusions	No data was excluded. All data that passed quality control parameters using standard sequencing and analysis algorithms (described in detail in the methods) were included in the analysis.
Replication	The in vitro experimental findings were reproduced in at least 3 independent experiments. The in vivo experimental findings were performed on 4-11 mice per group, the exact n value for each experimental findings is described in the Methods section or Figure legends of the manuscript. Each biological replicate was analyzed in separate independent experiments. Data shown in the figures represent the aggregate of all independent experiments, with Mean or Median as the center and SD, SEM, or interquartile range to define error bars.
Randomization	For the xenograft and bone marrow transplantation, the NSG or the B6.SJL mice were used as recipients, they were randomly distributed into different groups that also allowed the two sexes to be equally represented.<br>For primary mice phenotype analysis, or the transplantation, RNA-seq, ATAC-seq, ChIP-seq, 4C-seq anaysis based on primary mice BM LSK or LK cells, all the offsprings were genotyped at ~21days old, then littermates with right genotypes were all used for different experiment groups. For each experiments, age (±30 days) and sex matched were randomlly picked for analysis.<br>For all other mice used in this study, age (±30 days) and sex matched mice were always used, they were always randomlly picked when this restriction meets.<br>All other experiments perform in this study are all based on randomiization without selection bias.
Blinding	Majority of data was collected by automated software or by core facility staff members. Investigators were not be able to be blinded during data collection.<br><br>For data analysis, in cases where subjective views may influence data outcome (such as colony types, histology, paired daughter cell assay), researchers were not able to be blinded due to the obvious difference in experimental groups. However, the analysis of the same samples were performed by multiple researchers whose measurements reached consistent conclusions independently to rule out subjective bias.<br><br>For analysis carried out by automated softwares or unlikely to have subjective input (such as blood count, FACS, sequencing data analysis and qPCR), investigators were not blinded.

Behavioural & social sciences study design

All studies must disclose on these points even when the disclosure is negative.

Study description	*Briefly describe the study type including whether data are quantitative, qualitative, or mixed-methods (e.g. qualitative cross-sectional, quantitative experimental, mixed-methods case study).*
Research sample	*State the research sample (e.g. Harvard university undergraduates, villagers in rural India) and provide relevant demographic information (e.g. age, sex) and indicate whether the sample is representative. Provide a rationale for the study sample chosen. For studies involving existing datasets, please describe the dataset and source.*
Sampling strategy	*Describe the sampling procedure (e.g. random, snowball, stratified, convenience). Describe the statistical methods that were used to predetermine sample size OR if no sample-size calculation was performed, describe how sample sizes were chosen and provide a rationale for why these sample sizes are sufficient. For qualitative data, please indicate whether data saturation was considered, and what criteria were used to decide that no further sampling was needed.*
Data collection	*Provide details about the data collection procedure, including the instruments or devices used to record the data (e.g. pen and paper, computer, eye tracker, video or audio equipment) whether anyone was present besides the participant(s) and the researcher, and whether the researcher was blind to experimental condition and/or the study hypothesis during data collection.*

Timing	*Indicate the start and stop dates of data collection. If there is a gap between collection periods, state the dates for each sample cohort.*
Data exclusions	*If no data were excluded from the analyses, state so OR if data were excluded, provide the exact number of exclusions and the rationale behind them, indicating whether exclusion criteria were pre-established.*
Non-participation	*State how many participants dropped out/declined participation and the reason(s) given OR provide response rate OR state that no participants dropped out/declined participation.*
Randomization	*If participants were not allocated into experimental groups, state so OR describe how participants were allocated to groups, and if allocation was not random, describe how covariates were controlled.*

Ecological, evolutionary & environmental sciences study design

All studies must disclose on these points even when the disclosure is negative.

Study description	*Briefly describe the study. For quantitative data include treatment factors and interactions, design structure (e.g. factorial, nested, hierarchical), nature and number of experimental units and replicates.*
Research sample	*Describe the research sample (e.g. a group of tagged Passer domesticus, all Stenocereus thurberi within Organ Pipe Cactus National Monument), and provide a rationale for the sample choice. When relevant, describe the organism taxa, source, sex, age range and any manipulations. State what population the sample is meant to represent when applicable. For studies involving existing datasets, describe the data and its source.*
Sampling strategy	*Note the sampling procedure. Describe the statistical methods that were used to predetermine sample size OR if no sample-size calculation was performed, describe how sample sizes were chosen and provide a rationale for why these sample sizes are sufficient.*
Data collection	*Describe the data collection procedure, including who recorded the data and how.*
Timing and spatial scale	*Indicate the start and stop dates of data collection, noting the frequency and periodicity of sampling and providing a rationale for these choices. If there is a gap between collection periods, state the dates for each sample cohort. Specify the spatial scale from which the data are taken*
Data exclusions	*If no data were excluded from the analyses, state so OR if data were excluded, describe the exclusions and the rationale behind them, indicating whether exclusion criteria were pre-established.*
Reproducibility	*Describe the measures taken to verify the reproducibility of experimental findings. For each experiment, note whether any attempts to repeat the experiment failed OR state that all attempts to repeat the experiment were successful.*
Randomization	*Describe how samples/organisms/participants were allocated into groups. If allocation was not random, describe how covariates were controlled. If this is not relevant to your study, explain why.*
Blinding	*Describe the extent of blinding used during data acquisition and analysis. If blinding was not possible, describe why OR explain why blinding was not relevant to your study.*

Did the study involve field work? ☐ Yes ☐ No

Field work, collection and transport

Field conditions	*Describe the study conditions for field work, providing relevant parameters (e.g. temperature, rainfall).*
Location	*State the location of the sampling or experiment, providing relevant parameters (e.g. latitude and longitude, elevation, water depth).*
Access & import/export	*Describe the efforts you have made to access habitats and to collect and import/export your samples in a responsible manner and in compliance with local, national and international laws, noting any permits that were obtained (give the name of the issuing authority, the date of issue, and any identifying information).*
Disturbance	*Describe any disturbance caused by the study and how it was minimized.*

Reporting for specific materials, systems and methods

We require information from authors about some types of materials, experimental systems and methods used in many studies. Here, indicate whether each material, system or method listed is relevant to your study. If you are not sure if a list item applies to your research, read the appropriate section before selecting a response.

Materials & experimental systems

n/a	Involved in the study	
☐	☒	Antibodies
☐	☒	Eukaryotic cell lines
☒	☐	Palaeontology and archaeology
☐	☒	Animals and other organisms
☐	☒	Human research participants
☒	☐	Clinical data
☒	☐	Dual use research of concern

Methods

n/a	Involved in the study	
☐	☒	ChIP-seq
☐	☒	Flow cytometry
☒	☐	MRI-based neuroimaging

Antibodies

Antibodies used

All antibodies for FACS, ChIP and IHC were listed in Supplementary Table 7. The dilution used for each antibody was described in the methods section of the manuscript.

Validation

All the flow antibodies listed in Supplementary Table 7 were validated by BD Pharmingen and eBioscience company for FACS application, we also validated all these antibodies before flow analysis were performed.

Human CD45 (Abcam, Cat: ab10559) was validated by Abcam for IHC applications in their product statement, it was also validated by many references and my lab for IHC application.

MPO antibody (R&D, Cat: MAB3174) were previously validated through positive, negative, and isotype stainings by my lab for mice tissue IHC application.

H3K4me3 antibody (Millipore, Cat: 04-745) was validated by Millipore for ChIP-seq application.

SETD1A antibody (Bethyl, Cat: A300-289A) was validated by Bethyl for ChIP application.

LSD1 antibody (Millipore, Cat: 07-705) was validated by Millipore in their product statement for ChIP application.

MLL1 antbody (Novus Biologicals, Cat: NB600-248) was validated by my lab for ChIP application.

Catalog numbers and RRID of all antibodies were listed in the Supplementary Table 7.

Eukaryotic cell lines

Policy information about cell lines

Cell line source(s)

The AML cell lines OCI-AML3, MOLM13, NOMO1, SET-2, and OCI-AML2 were purchased from DSMZ (Leibniz Institute DSMZ-German Collection of Microorganisms and cell Cultures). The AML cell lines K-562, MV4-11, THP-1, and HEK293T cell line were purchased from ATCC. IMS-M2 cell line originated from (Chi et al., 2010, https://www.sciencedirect.com/science/article/pii/S0145212609004792).

Authentication

We used the cell lines directly from source; we performed morphologic examination; we validated the RNA overexpression, knockdown, knockout data with PCR or qPCR.

Mycoplasma contamination

All cell lines showed negative results upon MycoAlert™ Mycoplasma Detection Kit from Lonza.

Commonly misidentified lines
(See ICLAC register)

No commonly misidentified cell lines were used in the study.

Palaeontology and Archaeology

Specimen provenance

Provide provenance information for specimens and describe permits that were obtained for the work (including the name of the issuing authority, the date of issue, and any identifying information).

Specimen deposition

Indicate where the specimens have been deposited to permit free access by other researchers.

Dating methods

If new dates are provided, describe how they were obtained (e.g. collection, storage, sample pretreatment and measurement), where they were obtained (i.e. lab name), the calibration program and the protocol for quality assurance OR state that no new dates are provided.

☐ Tick this box to confirm that the raw and calibrated dates are available in the paper or in Supplementary Information.

Ethics oversight

Identify the organization(s) that approved or provided guidance on the study protocol, OR state that no ethical approval or guidance was required and explain why not.

Note that full information on the approval of the study protocol must also be provided in the manuscript.

Animals and other organisms

Policy information about studies involving animals; ARRIVE guidelines recommended for reporting animal research

Laboratory animals	HoxBlincTg transgenic mice, NPM1c+ KI mice and Setd1a KO mice are C57BL/6 background, both male and female mice are randomlly picked and used for experiments. All experiments based on mice samples utilized age- and sex- matched mice at the age of 2~12 months depending on the experiment needs.<br><br>For competitive transplantation assay, we used both male and female (randomly picked) B6.SJL mice at the age of 6~8 weeks. For Xenotransplantation, we used NOD-scid IL2Ry-null mice at the age of 6~8 weeks.<br><br>Mice housing condition is described in the method section of manuscript.
Wild animals	No wild animals were used in this study.
Field-collected samples	No field-collected samples were used in this study.
Ethics oversight	All studies were conducted in accordance with the regulatory guidelines by the Institutional Animal Care and Use Committee (IACUC) at the UT Health San Antonio and University of Miami Miller School of Medicine.

Note that full information on the approval of the study protocol must also be provided in the manuscript.

Human research participants

Policy information about studies involving human research participants

Population characteristics	Patients' characteristics are listed in Table S1.
Recruitment	All the AML patients received by the Institute of Hematology and Blood Disease Hospital, Tianjin, China for the first time from 10/2016 to 12/2016 were used to collect data in Figure1a without selection bias.<br><br>1315#, 921#, and LPP4# AML patients samples were used for PDX bone marrow transplantation. These samples were randomly picked from samples colllected by Pennsylvania State University College of Medicine once the samples meet the experimental requirements such as harboring genetic alterations such as NPM1c+ plus FLT3wt, NPM1c+ plus FLT3mu, or NPM1wt as indicated in Figure 1i, j, s2f-i. There is no selection bias for these human AML samples.
Ethics oversight	All human samples from healthy donors and patients with primary AML were obtained after informed consent following the guidelines of the Institutional Review Board of Pennsylvania State University College of Medicine (IRB protocol #2000-186 and #29252 EP) or the Institute of Hematology and Blood Disease Hospital, Tianjin, China.

Note that full information on the approval of the study protocol must also be provided in the manuscript.

Clinical data

Policy information about clinical studies

All manuscripts should comply with the ICMJEguidelines for publication of clinical research and a completedCONSORT checklist must be included with all submissions.

Clinical trial registration	*Provide the trial registration number from ClinicalTrials.gov or an equivalent agency.*
Study protocol	*Note where the full trial protocol can be accessed OR if not available, explain why.*
Data collection	*Describe the settings and locales of data collection, noting the time periods of recruitment and data collection.*
Outcomes	*Describe how you pre-defined primary and secondary outcome measures and how you assessed these measures.*

Dual use research of concern

Policy information about dual use research of concern

Hazards

Could the accidental, deliberate or reckless misuse of agents or technologies generated in the work, or the application of information presented in the manuscript, pose a threat to:

No	Yes	
☐	☐	Public health
☐	☐	National security
☐	☐	Crops and/or livestock
☐	☐	Ecosystems
☐	☐	Any other significant area

Experiments of concern

Does the work involve any of these experiments of concern:

No	Yes	
☐	☐	Demonstrate how to render a vaccine ineffective
☐	☐	Confer resistance to therapeutically useful antibiotics or antiviral agents
☐	☐	Enhance the virulence of a pathogen or render a nonpathogen virulent
☐	☐	Increase transmissibility of a pathogen
☐	☐	Alter the host range of a pathogen
☐	☐	Enable evasion of diagnostic/detection modalities
☐	☐	Enable the weaponization of a biological agent or toxin
☐	☐	Any other potentially harmful combination of experiments and agents

ChIP-seq

Data deposition

☒ Confirm that both raw and final processed data have been deposited in a public database such as GEO.

☒ Confirm that you have deposited or provided access to graph files (e.g. BED files) for the called peaks.

Data access links<br>*May remain private before publication.*	https://www.ncbi.nlm.nih.gov/geo/query/acc.cgi?acc=GSE115096
Files in database submission	1. GSM3165100, WT-H3K4me3-ChIP-seq;<br>2. GSM3165101, Hoxblinc-Tg-H3K4me3-ChIP-seq;<br>3. GSM4710563 , WT-MLL1-ChIP-seq-LSK;<br>4. GSM4710564, Hoxblinc-Tg-MLL1-ChIP-seq-LSK.
Genome browser session<br>(e.g. UCSC)	Integrated Genomic Viewer<br>http://software.broadinstitute.org/

Methodology

Replicates	None, just only once sequence.
Sequencing depth	Sequencing was carried out through paired-end 100bp platform.<br><br>WT-H3K4me3-CHIP-seq total reads: 29,774,596<br>WT-H3K4me3-CHIP-seq mapped reads: 27,133,299<br>HoxBlinc-Tg-H3K4me3 CHIP-seq total reads: 29,732,688<br>HoxBlinc-Tg-H3K4me3 CHIP-seq mapped reads: 26,667,210<br><br>WT-MLL1 CHIP-seq total reads: 32621325<br>WT-MLL1 CHIP-seq mapped reads: 30194298<br>HoxBlinc-Tg-MLL1 CHIP-seq total reads: 33012214<br>HoxBlinc-Tg-MLL1 CHIP-seq mapped reads: 30127431
Antibodies	Anti-H3K4me3 antibody, millipore, Cat: 04-745, RRID: AB_1163444.<br>Anti-KMT2A/MLL1 antibody, Novus Biologicals, Cat: NB600-248, RRID: AB_2145479
Peak calling parameters	Peak calling was performed using MACS algorithm (version 2.1.1):<br><br>macs2 callpeak -t WT-H3K4me3.bam -f BAM -g mm -n test -B -q 0.01<br>macs2 callpeak -t Hoxblinc-Tg-H3K4me3.bam -f BAM -g mm -n test -B -q 0.01<br><br>macs2 callpeak -t WT-MLL1.bam -f BAM -g mm -n test -B -q 0.01

	macs2 callpeak -t Hoxblinc-Tg-MLL1.bam -f BAM -g mm -n test -B -q 0.01
Data quality	FastQC was carried out to evaluate our sequencing data quality.<br>Parameter:<br>fastqc -f fastq -o result/ WT-H3K4me3.fastq.gz<br>fastqc -f fastq -o result/ Hoxblinc-Tg-H3K4me3.fastq.gz<br><br>fastqc -f fastq -o result/ WT-MLL1.fastq.gz<br>fastqc -f fastq -o result/ Hoxblinc-Tg-MLL1.fastq.gz
Software	cutadapt/1.18 program; Bowtie2/2.2.9; Deeptools/3.1.3; macs2/2.2.6; homer/4.10; Integrated Genomic Viewer (version 2.4.19)

Flow Cytometry

Plots

Confirm that:

- [x] The axis labels state the marker and fluorochrome used (e.g. CD4-FITC).
- [x] The axis scales are clearly visible. Include numbers along axes only for bottom left plot of group (a 'group' is an analysis of identical markers).
- [x] All plots are contour plots with outliers or pseudocolor plots.
- [x] A numerical value for number of cells or percentage (with statistics) is provided.

Methodology

Sample preparation	Total white blood cells were obtained after lysis of red blood cells with red blood cell lysis buffer (QIAGEN 1045722). Single-cell suspensions from BM, spleen and PB were stained with panels of fluorochrome-conjugated antibodies (listed in Table S7). The analyses were performed using BD LSRII or LSR Fortessa flow cytometer. All data were analyzed by FlowJo.V10 software. For FACS sorting, briefly, BM cells from 6-8 weeks old mice were pre-enriched with lineage depletion beads (MiltenyiBiotec, Bergisch Gladbach, Germany) and then stained with c-Kit, lineage, and Sca-1 antibodies (listed in Table S7, lineage flow antibody was diluted as 1:25, all other flow antibodies were diluted as 1:50) and then sorted by BD FACSAria . The purity of selected LSK cells were routinely over 98%.
Instrument	The FACS analyses were performed on BD LSR II or LSR Fortessa flow cytometer. The FACS sorting was performed on BD FACSAria II.
Software	BD FACSDIVA™ was used to collect data, FlowJo V10 was used to analyze data.
Cell population abundance	The abundance of relevant cells within post-sort fractions were over 98%, which was determined by re-run the flow analysis for the sorted cells and checking the percentage of the relevant cell under FSC/SSC gate.
Gating strategy	Isotype control was set to determine the gate of positive and negative populations.<br>For bone marrow, spleen and cell line samples, FSC/SSC gate was set to gate all cells in, but exclude small debris.<br>For blood samples, FSC/SSC gate was set to gate all karyocyte cells in, but exclude small debris and red blood cell residue.<br>Gating strategy is examplified by Table S9.

- [x] Tick this box to confirm that a figure exemplifying the gating strategy is provided in the Supplementary Information.

Magnetic resonance imaging

Experimental design

Design type	*Indicate task or resting state; event-related or block design.*
Design specifications	*Specify the number of blocks, trials or experimental units per session and/or subject, and specify the length of each trial or block (if trials are blocked) and interval between trials.*
Behavioral performance measures	*State number and/or type of variables recorded (e.g. correct button press, response time) and what statistics were used to establish that the subjects were performing the task as expected (e.g. mean, range, and/or standard deviation across subjects).*

Acquisition

Imaging type(s) | *Specify: functional, structural, diffusion, perfusion.*

Field strength | *Specify in Tesla*

Sequence & imaging parameters | *Specify the pulse sequence type (gradient echo, spin echo, etc.), imaging type (EPI, spiral, etc.), field of view, matrix size, slice thickness, orientation and TE/TR/flip angle.*

Area of acquisition | *State whether a whole brain scan was used OR define the area of acquisition, describing how the region was determined.*

Diffusion MRI ☐ Used ☐ Not used

Preprocessing

Preprocessing software | *Provide detail on software version and revision number and on specific parameters (model/functions, brain extraction, segmentation, smoothing kernel size, etc.).*

Normalization | *If data were normalized/standardized, describe the approach(es): specify linear or non-linear and define image types used for transformation OR indicate that data were not normalized and explain rationale for lack of normalization.*

Normalization template | *Describe the template used for normalization/transformation, specifying subject space or group standardized space (e.g. original Talairach, MNI305, ICBM152) OR indicate that the data were not normalized.*

Noise and artifact removal | *Describe your procedure(s) for artifact and structured noise removal, specifying motion parameters, tissue signals and physiological signals (heart rate, respiration).*

Volume censoring | *Define your software and/or method and criteria for volume censoring, and state the extent of such censoring.*

Statistical modeling & inference

Model type and settings | *Specify type (mass univariate, multivariate, RSA, predictive, etc.) and describe essential details of the model at the first and second levels (e.g. fixed, random or mixed effects; drift or auto-correlation).*

Effect(s) tested | *Define precise effect in terms of the task or stimulus conditions instead of psychological concepts and indicate whether ANOVA or factorial designs were used.*

Specify type of analysis: ☐ Whole brain ☐ ROI-based ☐ Both

Statistic type for inference (See Eklund et al. 2016) | *Specify voxel-wise or cluster-wise and report all relevant parameters for cluster-wise methods.*

Correction | *Describe the type of correction and how it is obtained for multiple comparisons (e.g. FWE, FDR, permutation or Monte Carlo).*

Models & analysis

n/a	Involved in the study
☐	☐ Functional and/or effective connectivity
☐	☐ Graph analysis
☐	☐ Multivariate modeling or predictive analysis

Functional and/or effective connectivity | *Report the measures of dependence used and the model details (e.g. Pearson correlation, partial correlation, mutual information).*

Graph analysis | *Report the dependent variable and connectivity measure, specifying weighted graph or binarized graph, subject- or group-level, and the global and/or node summaries used (e.g. clustering coefficient, efficiency, etc.).*

Multivariate modeling and predictive analysis | *Specify independent variables, features extraction and dimension reduction, model, training and evaluation metrics.*
